# Critical role of intestinal interleukin-4 modulating regulatory T cells for desensitization, tolerance, and inflammation of food allergy

**DOI:** 10.1371/journal.pone.0172795

**Published:** 2017-02-24

**Authors:** Haruyo Nakajima-Adachi, Kyoko Shibahara, Yoko Fujimura, Jun Takeyama, Erika Hiraide, Akira Kikuchi, Hitoshi Murakami, Akira Hosono, Tomonori Nochi, Yoshio Wakatsuki, Naoki Shimojo, Shuichi Kaminogawa, Ryuichiro Sato, Hiroshi Kiyono, Satoshi Hachimura

**Affiliations:** 1 Department of Applied Biological Chemistry, Graduate School of Agricultural and Life Sciences, The University of Tokyo, Tokyo, Japan; 2 Department of Microbiology and Immunology, Institute of Medical Science, The University of Tokyo, Tokyo, Japan; 3 Research Center for Food Safety, Graduate School of Agricultural and Life Sciences, The University of Tokyo, Tokyo, Japan; 4 Department of Food Bioscience and Biotechnology, Nihon University, Kanagawa, Japan; 5 Department of Clinical Bio-regulatory Science, Kyoto University Graduate School of Medicine, Kyoto, Japan; 6 The Department of Pediatrics, Chiba University, Chiba, Japan; 7 Core Research for Evolutional Science and Technology (CREST), Japan Science and Technology Agency, Tokyo, Japan; 8 International Research and Development Center for Mucosal Vaccines, Institute of Medical Science, The University of Tokyo, Tokyo, Japan; 9 Department of Medical Genome Science, Graduate School of Frontier Science, The University of Tokyo, Tokyo, Japan; 10 Graduate School of Medicine, The University of Tokyo, Tokyo, Japan; University of South Carolina School of Medicine, UNITED STATES

## Abstract

**Background and objective:**

The mechanism inducing either inflammation or tolerance to orally administered food allergens remains unclear. To investigate this we analyzed mouse models of food allergy (OVA23-3) and tolerance (DO11.10 [D10]), both of which express ovalbumin (OVA)-specific T-cell receptors.

**Methods:**

OVA23-3, recombination activating gene (RAG)-2-deficient OVA23-3 (R23-3), D10, and RAG-2-deficient D10 (RD10) mice consumed a diet containing egg white (EW diet) for 2–28 days. Interleukin (IL)-4 production by CD4^+^ T cells was measured as a causative factor of enteropathy, and anti-IL-4 antibody was used to reveal the role of Foxp3^+^ OVA-specific Tregs (aiTreg) in this process.

**Results:**

Unlike OVA23-3 and R23-3 mice, D10 and RD10 mice did not develop enteropathy and weight loss on the EW diet. On days 7–10, in EW-fed D10 and RD10 mice, splenic CD4^+^ T cells produced significantly more IL-4 than did those in the mesenteric lymph nodes (MLNs); this is in contrast to the excessive IL-4 response in the MLNs of EW-fed OVA23-3 and R23-3 mice. EW-fed R23-3 mice had few aiTregs, whereas EW-fed RD10 mice had them in both tissues. Intravenous injections of anti-IL-4 antibody recovered the percentage of aiTregs in the MLNs of R23-3 mice. On day 28, in EW-fed OVA23-3 and R23-3 mice, expression of Foxp3 on CD4^+^ T cells corresponded with recovery from inflammation, but recurrence of weight loss was observed on restarting the EW diet after receiving the control-diet for 1 month. No recurrence developed in D10 mice.

**Conclusions:**

Excessive IL-4 levels in the MLNs directly inhibited the induction of aiTregs and caused enteropathy. The aiTregs generated in the attenuation of T cell-dependent food allergic enteropathy may function differently than aiTregs induced in a tolerance model. Comparing the two models enables to investigate their aiTreg functions and to clarify differences between inflammation with subsequent desensitization versus tolerance.

## Introduction

Oral ingestion of food generally induces tolerance against food components [1], but in some cases, food intake causes excessive inflammatory responses that lead to food allergy [2]. The same orally administered allergen can induce either tolerance or inflammation, but the mechanisms that determine which response is induced remain unclear. Elucidating the mechanisms that underlie the shift between tolerance and inflammation will facilitate finding appropriate treatment options for food allergy, such as oral immunotherapy. However, clinical studies alone yield insufficient data for exploring these mechanisms, and traditional animal models are inappropriate for these purposes [3–5]. For example, in traditional models, adjuvants are used with food antigens to sensitize the animals; this practice fundamentally alters the immune responses of the mice and complicates direct analysis of the process establishing antigen-specific immune responses

In contrast to traditional models, OVA23-3 mice are appropriate as animal models for analyzing the mechanisms by which diverse, complex immune responses (i.e., tolerance, desensitization, and inflammation) are induced in response to orally administered ovalbumin (OVA); in this model, the processes by which intestinal inflammation and subsequent hyporesponsiveness to orally administered OVA alone are established can be assessed from the onset of sensitization and in the absence of any confounding influences due to an adjuvant [6, 7]. For example, feeding an egg-white-based diet (EW diet) to OVA23-3 mice initially induced severe enteropathy, produced weight loss, and increased serum OVA-specific IgE responses, whereas continued feeding of the EW was associated with amelioration of the inflammatory responses [6]. These previous experiments clarified that interleukin (IL)-4-producing, OVA-specific CD4^+^ T cells in the mesenteric lymph nodes (MLNs) caused the intestinal inflammation, in EW-fed OVA23-3 mice. Furthermore, continued feeding of the mice with the EW diet induced hyporesponsiveness of OVA-specific T cells, and thus resolution of severe inflammation; however, CD4^+^ T cells in the MLNs retained the ability to produce IL-4 during the recovery phase. Therefore, the hyporesponsiveness induced in OVA23-3 mice by prolonged feeding of the EW diet might not represent a tolerant phase of the inflammation but rather a desensitized phase, because persistent IL-4 production in the MLNs might continue to provoke intestinal inflammatory responses [7]. During desensitization, inflammatory responses are quiescent but recur when food allergens are re-administered after the first recovery from severe inflammation. Our previous results showed that examining the EW-fed OVA23-3 model alone was insufficient to clarify the mechanisms underlying the complex immune responses to oral administered OVA.

DO11.10 (D10) mice are known as a tolerance model in response to orally administered OVA [8]. The mice are on a BALB/cA genetic background and express different OVA-specific T-cell receptor (TCR) genes than OVA23-3 mice. Because they recognize different epitopes on the OVA323-339 peptide, the naïve CD4^+^ T cells of the two models exhibit different responses (Th2: OVA23-3 mice; Th1: D10 mice) [9], but the transfer of Th2-biased D10 OVA-specific CD4^+^ T cells could induce IgE production in EW-fed recipient BALB/cA mice [10]. Consistent with mechanisms for inducing oral tolerance [11], the OVA-specific CD4^+^ T cells from D10 mice may promote mild Th2 responses and subsequently induce IL-10 production or allergen-specific Foxp3^+^ CD4^+^ T cells (aiTregs) in response to oral administered OVA.

Although tolerance to orally administered food allergens is thought to be regulated by aiTregs [12], whether the aiTregs induced through continuous feeding of food allergens under conditions of excess IL-4 in the MLNs (e.g., in our EW-fed OVA23-3 mouse model) can attenuate food allergic intestinal inflammation remains unknown. A recent report indicates that IL-4 production inhibits the induction of intestinal aiTregs in mice carrying an IL-4Rα chain mutation (i.e., inactivation of immunoreceptor tyrosine-based inhibition motif); this mouse model develops food allergic anaphylaxis after sensitization with OVA and the adjuvant staphylococcal enterotoxin B [13]. In addition, aiTregs reprogrammed by excessive responses to IL-4 aggravate food allergic anaphylaxis through signals from FcεRI expressed on mast cells. In our OVA23-3 system, the enteropathy observed in EW-fed OVA23-3 mice is not dependent on IgE responses, but on excessive IL-4 production by OVA-specific CD4^+^ T cells [7]; therefore, in the OVA23-3 model of T-cell-dependent food allergic enteropathy, aiTregs may influence both the aggravation of and recovery from the food-induced inflammation.

In the present study, we compared phenotypes of OVA23-3 and D10 mice to establish a system that clarifies the mechanism causing the diverse immune responses to orally administered allergens. Unlike OVA23-3 mice, D10 mice did not exhibit any weight loss or enteropathy after consuming an EW-diet. In addition, the level of IL-4 in the MLNs—independent of systemic IL-4 production—influenced the induction of aiTregs in both EW-fed strains. In the current study, we describe how the D10 and OVA23-3 strains together comprise a system that enables us to investigate whether tolerance or intestinal inflammation predominates in response to oral administered OVA.

## Methods

### Ethics statements

We followed the ARRIVE guidelines and those of the University of Tokyo regarding the care and use of animals (approval no. P11-533, P15-22 and P15-23) when performing and reporting the present experiments and submitting this manuscript. All aspects of the experiments, including euthanasia, were approved by the Animal Use Committee of the Faculty of Agriculture at the University of Tokyo, in which some veterinarians are included. When necessary for cellular and histological analysis, mice were euthanized via cervical dislocation by experts. Our experiments were approved without setting preemptive humane endpoints. Although the mice developed weight loss and enteropathy by day 10 on the EW diet, they did not exhibit other clinical signs and recovered from the inflammation upon continued feeding of the diet for a total of 28 days. In addition, we incorporated measures to minimize distress as mentioned in the following section.

### Mice

OVA23-3 mice and recombination activating gene (RAG)-2-deficient OVA23-3 mice (R23-3 mice) on a BALB/cA genetic background were kindly provided by S. Habu (Tokai University School of Medicine) [14]. Heterogenic OVA23-3 mice and D10 mice backcrossed to BALB/cA mice obtained from CLEA Japan, Inc. (Tokyo, Japan) and RAG-2-deficient D10 (RD10) mice provided by Y. Wakatsuki were maintained in a specific-pathogen-free-room at the University of Tokyo. Guidelines formulated by the University of Tokyo were followed for the care and use of animals, including sterilized deionized drinking water, sterilized commercial chow, room temperature of 22°C, and a 12:12-h light:dark cycle. These conditions were monitored daily. Each cage (182 × 260 × 128 mm, [model. CL-0103-2, CLEA Japan, Inc.]) contained five or fewer mice.

### Ovalbumin administration

OVA23-3, R23-3, D10, and RD10 mice (age, 6 to 8 weeks) were fed with EW diet (Funabashi Farm Co.,Ltd. Chiba, Japan), or control diet (casein diet [Funabashi Farm], or CE2 diet, [CLEA Japan, Inc.]) for 28 days. The compositions of the diets have been described previously [6]. In addition, we have previously demonstrated similar results when using either casein or CE2 diet as a control diet [6]. To examine the effects of the regulatory activity at day 28 in EW-fed OVA23-3 and D10 mice, they were fed the EW diet for 28 days and then were given the control-diet for 1 month, after which they were switched back to the EW diet. Mice were weighed every 2 to 7 days. At the end of each experiment, the mice were euthanized and their tissues harvested for further analysis. Sera were stored at –80°C. Total serum immuoglobulin (Ig) E and OVA-specific IgE, IgG1, and IgG2a levels were analyzed by enzyme-linked immunosorbent assay, as described previously [6].

### Cell culture

MLN and splenic CD4^+^ T cells were positively purified from lymphocytes pooled from each mouse strain fed with the control-diet or EW diet by using MACS CD4 beads and an Auto MACS cell separation system (Miltenyi Biotec, Bergish Gladbach, Germany). According to flow cytometry (LSR model, BD Bioscience, Franklin Lakes, NJ, USA), the CD4^+^ T cell ratio exceeded 95% in each analysis. T–cell proliferation assays were performed in 96-well flat-bottom plates. CD4^+^ T cells (1 × 10^5^ cells/well) were cultured in the absence or presence of OVA (1 mg/mL) or in different concentrations of OVA (0.02–20 mg/mL) with mitomycin-C–treated splenocytes from BALB/cA mice as antigen-presenting cells (2–4 × 10^5^ cells/well) in complete RPMI1640. Proliferation and cytokine production were measured as described previously [7]. Briefly, for measurement of cytokine concentrations, culture supernatants of CD4^+^ T cells from RAG-2-deficient TCR-transgenic mice and OVA23-3 mice fed with the control-diet or EW diet were collected at 48 hours of culture, and those from D10 mice fed with both diets were collected at 72 hours. Proliferation of the cells in response to OVA was measured under similar cell culture conditions, at which we can obtain high response.

To analyze Foxp3 expression in cultured CD4^+^ T cells, naïve CD4^+^ T cells (1 × 10^5^ cells/well) from the spleens of RD10 and R23-3 mice were stimulated with OVA (0–10 mg/mL) in the presence of antigen-presenting cells (4 × 10^5^ cells/well) in 96-well flat-bottom plates; antigen–presenting cells were prepared by using the MACS system to remove CD4^+^ T cells from splenocytes of BALB/cA mice. After 72 hours, Foxp3 expression by the CD4^+^ T cells in each well was analyzed by flow cytometry. In stimulation with antibodies of OVA-specific CD4^+^ T cells, anti-CD3 monoclonal antibody (mAb) (50, 10, 2, 0.4, 0.1, 0.02, and 0 μg/mL; 145-2C11, BD Pharmingen) and anti-CD28 mAb (5 μg/mL; 37.51, BD Pharmingen) were coated on 96-well flat-bottomed plates at 4°C overnight. The supernatant was removed, and CD4^+^ T cells (1 × 10^5^ cells in 200 μL complete RPMI per well) of RD10 and R23-3 mice purified by MACS CD4 beads were combined with antigen-presenting cells (mitomycin-C–treated splenocytes from BALB/cA mice) and incubated for 24 hours. The supernatants were collected for determination of the cytokine levels. After removal of the culture supernatants of the cells, [^3^H]-thymidine (1 μCi; Moravek Biochemicals, Brea, CA, USA) was pulsed into the wells, for measurement of T-cell proliferations [7].

### Cytokine measurement

Cytokine concentration was determined by enzyme-linked immunosorbent assay, as described previously [15]. The pairs of capture mAbs and biotinylated secondary detection mAbs were as follows: BVD4-1D11 and BVD4-24G2 for IL-4, R4-6A2 and XMG1.2 for interferon (IFN)-γ, and JES6-1A12 and JES6-5H4 for IL-2 (all from BD Pharmingen). Supernatants from splenic CD4^+^ T cells of OVA23-3 mice were used as IL-2 standards, and purified IL-4 (PeproTech, Rocky Hill, NJ, USA) and IFN-γ (PeproTech) proteins were used for drawing standard curves.

### Flow cytometry

For flow cytometric analysis, single-cell suspensions prepared from spleen and MLNs or the cells collected from each well of 96-well flat-bottomed culture plates were stained in the presence of anti-CD16/CD32 mAbs (BD Pharmingen). To examine the surface molecules of CD4^+^ T cells, further staining was performed with the following mAbs; FITC-conjugated anti-mouse CD4 mAb (H129.19, BD Pharmingen), allophycocyanin-conjugated rat anti-mouse CD4 mAb (GK1.5, Biolegend, San Diego, CA, USA), Per-CP Cy5.5-conjugated rat anti-muse CD44 (IM7, Biolegend) and allophycocyanin-conjugated rat anti-mouse CD62L (MEL14, Biolegend). Intracellular staining of Foxp3 was performed by using PE mouse/rat Foxp3 staining sets (eBioscience, San Diego, CA, USA. The profiles were analyzed by flow cytometry (BD Caliber and BD FACSVerse; BD Biosciences) using FlowJo software (Tree Star, Ashland, OR, USA).

### Histologic analysis

Histologic analysis of enteropathy by hematoxylin and eosin (H&E) staining was performed as described previously [6]. The ratio of villous height to crypt depth was analyzed as typical and quantitative parameter of enteropathy induced in EW-fed OVA23-3, D10, R23-3, and RD10 mice as described previously [6]. Villous height and crypt depth were measured in ten randomly selected villi per mouse.

### Injection of mice with anti-IL-4 antibody

To investigate the ability of IL-4 in inducing the expression of Foxp3 molecules on the CD4^+^ T cells of EW-fed R23-3 mice, R23-3 mice were injected intravenously with anti-IL-4 mAb (1 mg; clone 11B11) or control antibody (1 mg; rat IgG, Cappel, Cochranville, PA, USA) on the day before and on day 3 of receiving the diet. Mice were weighed daily for 7 days. On day 7, the mice were euthanized, and their MLN and spleen cells and intestinal tissues were harvested. The expression of Foxp3 molecules on splenic and MLN CD4^+^ T cells was analyzed by flow cytometry.

### Statistical analysis

Results are presented as mean ± 1 standard deviation. The Mann–Whitney U test was used for analysis of statistical differences of serum antibody production and the Student’s t-test was used for other analysis between 2 groups (Microsoft Excel 2008, SSRI, Tokyo, Japan). Differences were considered statistically significant, when the *p* value was less than 0.05.

## Results

### D10 mice fed EW diet did not exhibit inflammatory responses

To obtain models for comparing the induction of inflammation or tolerance after feeding of a food allergen in the absence of an adjuvant or immunomodulatory molecules, we first examined whether D10 mice fed the EW diet for 10 or 28 days developed food allergic inflammation. These schedules corresponded to the phase of severe inflammation (days 7–10 of the EW diet) and recovery from inflammation (day 28 of the EW diet) observed in EW-fed OVA23-3 mice.

**Fig 1:**
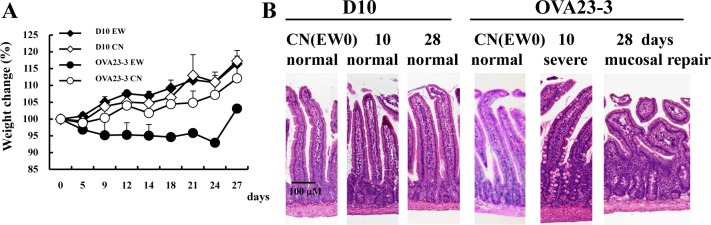
Lack of weight loss and enteropathy in EW-fed D10 mice. **A**, Weight change (relative to day 0 [100%]). The loss was solely indicated in EW-fed OVA23-3 group. **B**, H&E-stained histology of the jejunum of mice fed with control diet (CN) or fed with EW diet (EW) for 10 or 28 days. Severe inflammation (crypt elongation, villous atrophy, thickness of muscular layer, goblet cell hyperplasia) was present in day 10 of EW-fed OVA23-3 mice but not in that of EW-fed D10 mice (10 days). Furthermore, EW-feeding of OVA23-3 mice for 28 days (28 days) shows severe villous blunting, indicating moderate degree of inflammation under mucosal repair. These results are representative of three independent experiments using CN-fed D10 mice (n = 4), EW-fed D10 mice (n = 4) CN-fed OVA23-3 mice (n = 2), and EW-fed OVA23-3 mice (n = 2).

Compared with control-diet-fed D10 mice on days 7–10, EW-fed D10 mice had no weight loss or severe inflammation in their small intestine; in contrast OVA23-3 mice showed marked weight loss and severe small-intestinal inflammation at the same point (Fig 1A and 1B). On day 28, OVA 23–3 mice had regained some of the weight lost during the inflammatory phase, but their weight did not match that of the control-diet-fed mice, suggesting that inflammatory responses continued in the MLNs [7]. Throughout the experimental period, the inflammatory responses observed in the EW-fed OVA23-3 mice were similar to those of the EW-fed R23-3 mice, but neither EW-fed D10 mice nor EW-fed RD10 mice developed inflammation. (S1 Text and S1 Fig). R23-3 and RD10 mice have only OVA-specific CD4^+^ T cells as peripheral T cells because the *Rag2* knockout inhibits maturation of endogenous T cells. Therefore, the current results from the EW-fed R23-3 and RD10 mice confirm the critical role of OVA-specific CD4^+^ T cells in the development of inflammation in EW-fed OVA23-3 mice [7] and the loss of the ability of OVA-specific CD4^+^ T cells to induce inflammation in D10 mice.

**Fig 2:**
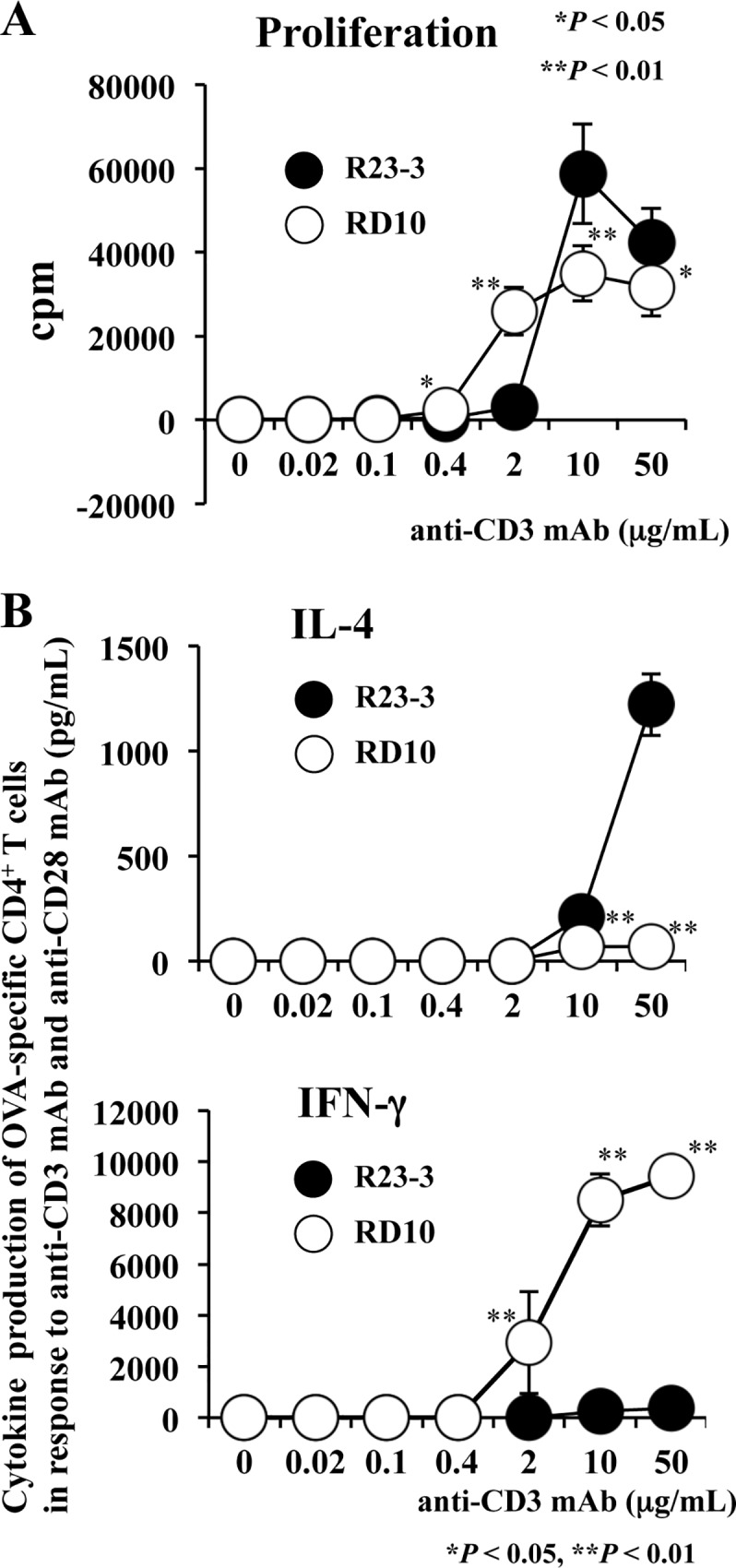
Proliferation and cytokines production of naïve OVA-specific CD4^+^ T cells from R23-3 and RD10 mice. Naïve OVA-specific CD4^+^ T cells purified from R23-3 (●) and RD10 (○) mice (n = 3 each) were stimulated with both anti-CD3 mAb and anti-CD28 mAb. **A**, Proliferation of OVA-specific CD4^+^ T cells. **B,** IL-4 (upper panel) and IFN-γ (lower panel) production in culture supernatants of OVA-specific CD4^+^ T cells. These results are representative of two independent experiments; *, *P*< 0.05; **, *P* < 0.01.

Next, in both strains, we investigated the phenotypes of OVA-specific CD4^+^ T cells after intracellular signaling from TCRs stimulated by anti-CD3 and anti-CD28 mAbs. The cytokine production profile of the cells from RD10 mice indicated a Th1 phenotype involving marked IFN-γ production, whereas that from R23-3 mice showed an apparent Th2 phenotype, with the production of large amounts of IL-4 (Fig 2). Similar results were obtained by using OVA323-339 peptide [9] and OVA (S2 Fig). However, the CD4^+^ T cells of R23-3 mice showed decreased production of IL-4 and increased levels of IFN-γ at high concentrations of OVA (S2 Fig). The elevated Th1 response after exposure to high OVA concentrations may result due to be negative feedback on the Th2 response by strong TCR stimulation [9]. In contrast, the increased stimulation due to the high dose of OVA achieved by feeding the EW diet in vivo stimulated the TCRs of naïve CD4^+^ T cells of OVA23-3 mice and the production of large amounts of IL-4, thereby leading to the allergic inflammatory responses in these mice. The lack of excessive Th2 responses in D10 CD4^+^ T cells stimulated through TCRs may correspond to the lack of intestinal inflammation in D10 mice. However, the IgG1 production of D10 mice at days 10 and 28 of EW diet but not at day 0 of the EW (CN) diet (S2 Text and S3 Fig) suggest that the EW diet might cause OVA-specific CD4^+^ T cells to exhibit a week Th2 response, in contrast to the Th1 response shown during in vitro culture (Fig 2 and S2 Fig). These results suggest that EW-feeding caused Th2 response in both strains, even if the level of Th2 responses differed between them, which might be induced by the differences between original characteristics of naïve CD4^+^ T cells of Th2 in R23-3 and of Th1 in RD10 mice.

### Systemic Th2 responses of OVA-specific CD4^+^ T cells in EW-fed D10 mice do not induce an inflammatory response

**Fig 3:**
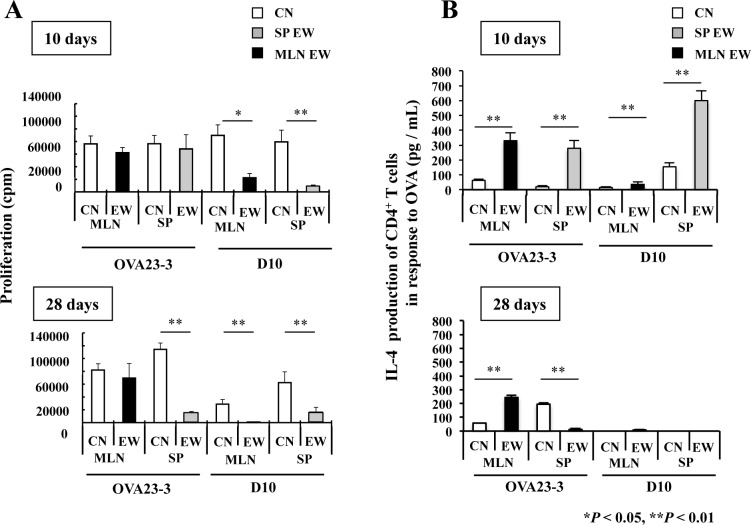
Responses of systemic or intestinal OVA-specific CD4^+^ T cells from EW-fed OVA23-3 and D10 mice. Proliferation and IL-4 production of OVA-specific CD4^+^ T cells purified from spleen (SP) or mesenteric lymph nodes (MLNs) of control-diet-fed (CN, n = 3) or EW-fed (n = 2 or 3) OVA23-3 or D10 mice (n = 3 or 4). **A,** Proliferation of OVA-specific CD4^+^ T cells on day 10 (upper panel) and day 28 (lower panel) of EW diet. **B,** IL-4 production of OVA-specific CD4^+^ T cells on day 10 (upper panel) and day 28 (lower panel) of EW diet. The CD4^+^ T-cell responses from the MLN (black bars) and spleen (SP; gray bars) of EW-fed mice and control-diet-fed mice (white bars) are shown. These results are representative of three independent experiments; *, *P* < 0.05; **, *P* < 0.01.

To examine the responses of CD4^+^ T cells in systemic or intestinal tissues to the EW diet, we first compared the time-dependent influence of the EW diet on CD4^+^ T cell function in each tissue between D10 and OVA23-3 mice. In the present study, we also examined RD10 and R23-3 mice as well as D10 and OVA23-3 mice; because RAG-2-deficient mice lack B cells, we thus examined in vivo OVA-specific CD4^+^ T cell functions without the confounding effect of antibody responses. On day 10 after the onset of the EW diet, CD4^+^ T cells from the spleen and MLNs of EW-fed OVA23-3 mice proliferated at similar levels as those from control-diet-fed mice (Fig 3A, OVA23-3 in upper panel), in agreement with previous results [7]. In contrast, the proliferation of CD4^+^ T cells from the MLNs of EW-fed D10 mice was decreased significantly compared with that of CD4^+^ T cells from control-diet-fed mice (Fig 3A, D10 in upper panel); however, the inhibition of proliferation in splenic CD4^+^ T cells from EW-fed compared with control-diet-fed D10 mice was not always apparet the day-10 time point (data not shown). However, by day 28 of the EW diet, in both D10 and OVA23-3 mice, the proliferative responses of splenic CD4^+^ T cells were attenuated compared with those of the control-diet-fed counterparts, showing hyporesponsiveness of CD4^+^ T cells (Fig 3A; D10 and OVA23-3 in lower panel, *P* < 0.01) [7]. The proliferation of MLN CD4^+^ T cells was significantly lower in EW-fed D10 mice than in the control-diet group (Fig 3A; D10 MLN-EW in lower panel, *P* < 0.01), whereas the proliferation of MLN CD4^+^ T cells did not decrease significantly in EW-fed OVA23-3 mice (Fig 3A; OVA23-3 MLN-EW, black bar in lower panel).

To confirm the responses of purified OVA-specific CD4^+^ T cells to the EW diet, we analyzed the T-cell responses of RD10 and R23-3 mice. On day 7 of the inflammatory phase, the proliferative responses of MLN CD4^+^ T cells from EW-fed RD10 mice were attenuated unlike those of R23-3 mice (S4A Fig, R23-3 and RD10 MLN, black bar in left panel). These responses of R23-3 and RD10 mice were similar to those of EW-fed OVA23-3 and D10 mice (Fig 3A), respectively. On day 28, the proliferation of MLN CD4^+^ T cells from EW-fed R23-3 and RD10 mice was attenuated (S4A Fig, R23-3 and RD10 MLN, black bar in right panel), although splenic OVA-specific CD4^+^ T cells were inconsistently hyporesponsive (data not shown). Why the proliferation of splenic T cells in RAG-2-deficient mice is not always attenuated remains unknown; however the EW diet promoted persistent, marked systemic responses in D10 mice, whereas IL-2 production by splenic CD4^+^ T cells from OVA23-3 and D10 mice (S4B Fig, gray bars in left panel) and those from ROVA2-3 and RD10 mice (S4B Fig, gray bars in right panel) was significantly inhibited compared with that of control-diet-fed counterparts *(P* < 0.01 or 0.05), as seen for the MLN responses in both strains (S4B Fig; black bars in left and right panels, *P* < 0.01 or 0.05).

Next, we analyzed CD4^+^ T cell function during EW feeding. On day 10, IL-4 production by both MLN and splenic CD4^+^ T cells of EW-fed OVA23-3 mice was significantly increased compared with that of control-diet-fed mice (Fig 3B; OVA23-3 in upper panel, *P* < 0.01). The IL-4 responses in the MLNs were slightly higher in the EW-fed D10 mice than in control-diet-fed mice (Fig 3B; D10, black bar in upper panel, *P* < 0.01), whereas the IL-4 level from control-diet-fed D10 mice was lower than that of control-diet-fed OVA23-3 mice (data not shown, *P* < 0.01), and the IL-4 responses in the spleens of EW-fed D10 mice were inconsistent (data not shown). The production of IL-4 by the splenic CD4^+^ T cells of EW-fed OVA23-3 mice on day 28 was markedly suppressed compared with that on day 10 (Fig 3B; OVA23-3, gray bar in lower panel, *P* < 0.01); however, in the MLNs of OVA23-3 mice or in the spleens of D10 mice, IL-4 production was not always inhibited (Fig 3B; lower panel). The results of IL-4 production obtained from both RD10 and R23-3 mice on day 7 (S5A Fig, upper panel) and 28 day (S5A Fig, lower panel) of EW diet were similar to those from their *Rag2*^+^ counterparts. In addition, the spleen cells from RD10 mice demonstrated inconsistent responses in IFN-γ production on day 28 of EW-feeding (data not shown), while those from EW-fed R23-3 mice produced significant higher production of IFN-γ than those from control-diet-fed the mice (S5B Fig, R23-3, gray bar in lower panel, *P* < 0.05). IFN-γ production of MLN CD4^+^ T cells from EW-fed R23-3 mice showed significant decrease than those from control-diet-fed the mice (S5B Fig, R23-3, black bar in lower panel, *P* < 0.05) on day 28. These results suggested that, 1), in contrast to the persistence of the Th2 inflammatory responses in the MLNs and their attenuation in the spleens of EW-fed OVA23-3 mice, the EW diet induced systemic Th2 responses in D10 mice. 2), IFN-γ production by naïve CD4^+^ T cells at high concentration of OVA in R23-3 mice (Fig 2 and S2 Fig) may not lead to attenuation of the enteropathy of EW-fed R23-3 mice on day 28, because of the significant decrease of IFN-γ production by MLN CD4^+^ T cells, but higher responses of IFN-γ by splenic CD4^+^ T cells may contribute to systemic regulation of inflammatory responses on day 28 of EW diet.

### Early systemic Th2 responses induced in EW-fed D10 mice are insufficient to induce food allergic inflammation

**Fig 4:**
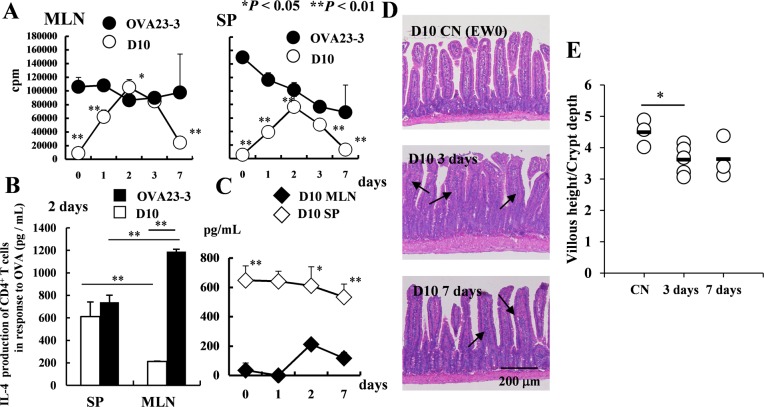
CD4^+^ T-cell function after shorter period of EW feeding in D10 and OVA23-3 mice. **A**, Proliferative responses on days 0 through 7 and **B**, IL-4 levels on day 2 of CD4^+^ T cells purified from MLNs or spleen (SP) of EW-fed OVA23-3 (n = 3) and D10 mice (n = 3). **C**, IL-4 production by CD4^+^ T cells from spleen (SP) and MLNs of EW-fed D10 mice on days 0 through 7. **D**, Jejual sections obtained from D10 mice fed with EW diet for 0 days (top panel; D10 CN[EW0]), 3 days (middle panel; D10 3 days), and 7 days (bottom panel; D10 7 days). Arrows indicate regions of cellular infiltration into the lamina propria of each villus in sections from EW-fed D10 mice. The top panel indicates the normal jejunum of CN-fed D10 mice; the jejunal sections from EW-fed D10 mice on day 3 (middle panel) and day 7 (bottom panel) show slight significant elongation of crypts (described in **E**) compared with normal section (top panel). **E,** Ratio of villous height to crypt depth of the jejunum. The ratio was significantly decreased (that is, crypt elongation; *P < 0.05) in EW-fed group (3 days) compared with control groups. The results shown are representative of the 2 replicates. Supernatants were collected at 48 hours after stimulation for OVA23-3 mice or after 72 hours for D10 mice.

To clearly investigate the activation of systemic and intestinal CD4^+^ T cells from D10 mice, we evaluated the functions of CD4^+^ T cells at earlier experimental time points of 0, 2, 3, and 7 days from the onset of the EW diet. For the proliferative responses of CD4^+^ T cells purified from the spleens and MLNs of the two EW-fed strains until day 7 (Fig 4A), the level of both systemic and intestinal D10 CD4^+^ T cell proliferation peaked on days 2 to 3 and began to decrease on day 7. The level of IL-4 produced by splenic CD4^+^ T cells of D10 mice on day 2 was comparable to that produced by those of OVA23-3 mice (Fig 4B). However, the production of IL-4 by the MLN CD4^+^ T cells in D10 mice never reached that achieved in OVA23-3 mice (Fig 4B). Furthermore, throughout the early time period, the IL-4 level of splenic CD4^+^ T cells from EW-fed D10 mice was significantly higher than that of their MLNs (Fig 4C; day 2, *P* < 0.05; days 0 and 7, *P* < 0.01). On days 2 or 3 and day 7, sections of the jejunum from D10 mice exhibited only minor inflammatory changes. In comparison with CN-fed D10 mice, the ratio of villous height to crypt depth showed significant decrease (Fig 4E) and cell infiltration into the lamina propria was increased in jejunal sections from EW-fed D10 mice (Fig 4D), presumably due to the transiently increased IL-4 production in the MLNs (Fig 4C). These results confirm that, although D10 mice immediately developed strong systemic Th2 responses when they were fed the EW diet, they were sufficient to produce significantly higher serum OVA-specific IgG1 levels than those in OVA23-3 mice (*P* < 0.05, S2 Text and S3 Fig). In addition, the decreased IL-4 production in the MLNs of EW-fed D10 mice might induce minor Th2 responses and promote regulatory responses and the acquisition of tolerance to OVA.

### IL-4 production in systemic and intestinal tissues plays different roles in the induction of aiTregs in both strains of mice

**Fig 5:**
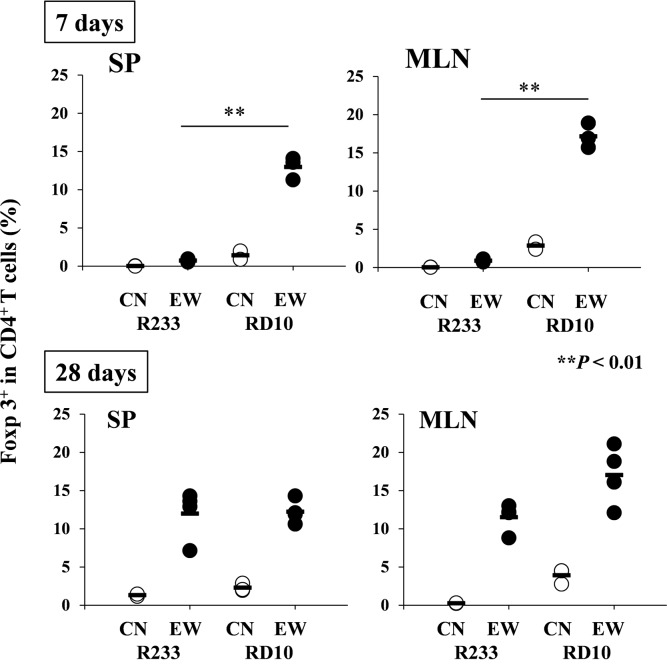
Induction of OVA-specific Foxp3^+^ CD4^+^ T cells in EW-fed R23-3 and RD10 mice. The percentage of OVA-specific Foxp3^+^ CD4^+^ T cells per OVA-specific CD4^+^ T cells in R23-3 and RD10 mice during continuous EW feeding is indicated. SP: spleen; MLN: mesenteric lymph node; RD10: RAG-2-deficient D10 mice; R23-3: RAG-2-deficient OVA23-3 mice; CN: control diet group; EW: egg-white diet group. These data are representative of two independent experiments. For day 7, R23-3 CN: n = 2; EW: n = 3; RD10 CN: n = 2; EW: n = 3. For day 28, R23-3 CN: n = 2; EW: n = 4; RD10 CN: n = 3; EW: n = 4. **, *p* < 0.01.

Using R23-3 and RD10 mice, we then examined the relationship between the induction of aiTreg cells and IL-4 production by T cells, to elucidate differences between the two models in the induction of either inflammation or tolerance in response to an EW diet. RAG-2-deficient OVA TCR-transgenic mice lack natural Tregs and can only develop aiTregs after sensitization with OVA. In OVA-specific CD4^+^ T cells of control-diet-fed R23-3 mice, cells expressing Foxp3 molecules were negligible, but these populations were slightly increased on day 7 (that is, during the inflammatory stage) of the EW diet (averages; 0.75% in spleen and 0.9% in MLNs) (Fig 5; upper panel and S6 Fig; R23-3, EW 7 days). On day 28 of continued EW feeding and thus induced hyporesponsiveness of CD4^+^ T cells (Fig 3A; lower panel), the percentage of aiTregs per total CD4^+^ T cells was increased in both spleens and MLNs relative to day-7 levels, and the proportions were comparable between tissues (averages; 12.0% in spleen and 11.5% in MLNs of R23-3; Fig 5, lower panel and S6 Fig; R23-3, EW 28 days). The percentage of Foxp3^+^ cells in OVA-specific CD4^+^ T cells of EW-fed RD10 mice in both tissues were higher than those of EW-fed R23-3 mice on day 7 of the experimental period, and the percentage was greater in the MLNs (averages; 17.1%) than in the spleen (averages; 13.0%), suggesting that regulatory responses were higher in MLNs than in the spleen of EW-fed RD10 mice (Fig 5, upper panel, and S6 Fig; RD10, EW 7 days). The higher percentage of aiTregs in the MLNs (averages; 17.0%) than in the spleen (averages; 12.2%) continued until day 28 (Fig 5, RD10 in lower panel, and S6 Fig; RD10, EW 28 days). In EW-fed OVA23-3 mice, the percentages of Foxp3^+^ CD4^+^ T cells in both MLNs and spleen increased from the inflammatory to the tolerant phase (S7 Fig). Throughout the 28-day experimental period, the proportion of Foxp3^+^ CD4^+^ T cells was significantly higher in the spleen than in the MLNs (S7 Fig, EW 0 and 10 days, *P* < 0.01, EW 28 days, *P* < 0.05). However, the percentage of Foxp3^+^ CD4^+^ T cells in the MLNs of the OVA23-3 mice fed the control-diet was 60% of those cells in the spleen. In addition, the percentage of Foxp3^+^ CD4^+^ T cells in the MLNs of the EW-fed mice decreased to 50% of the cells in the spleen on day 10 but increased to 80% on day 28; these data strongly indicate that, according to the attenuation of T cell activation throughout the experimental period, regulatory responses were intensified in the MLNs, whereby aiTregs affected the recovery from the inflammation (S3 Text and S7 Fig). These results suggest that, in EW-fed OVA23-3 and R23-3 mice, excessive IL-4 production inhibited the induction of aiTreg during T-cell-dependent inflammation, thus aggravating and establishing food allergic enteropathy. In EW-fed RD10 mice, the lack of weight loss and the increased aiTreg ratios in both the spleen and MLNs suggest that 1) systemic Th2 responses did not greatly influence aiTreg induction, and 2) splenic aiTregs might contribute to the maintenance of systemic hyporesponses to orally administered OVA in cooperation with increased regulatory responses in MLNs.

**Fig 6:**
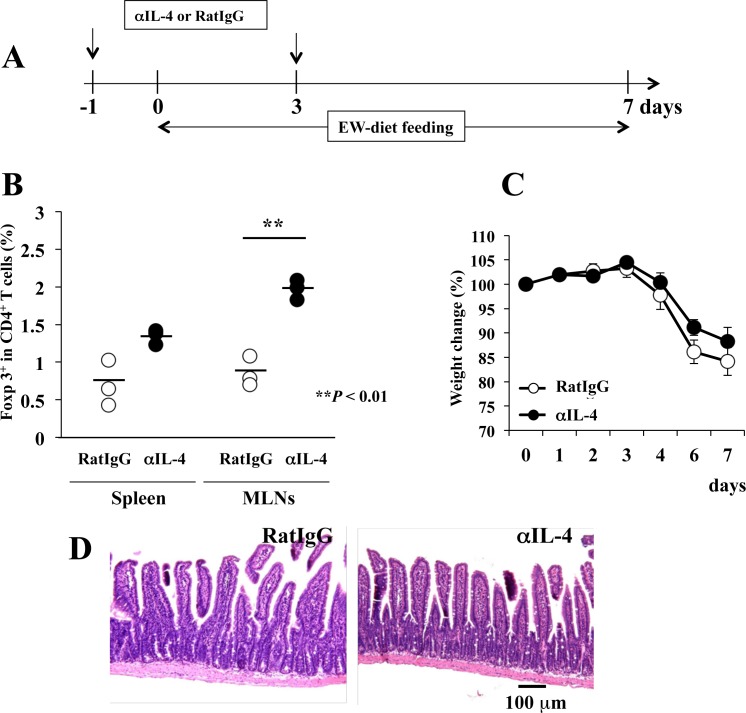
Treatment with anti-IL-4 mAb upregulates Foxp3 expression on MLN OVA-specific CD4^+^ T cells of EW-fed R23-3 mice. **A,** Protocol for anti-IL-4 mAb (αIL-4) or control antibody (RatIgG) injection (↓) and EW diet. **B,** Percentages of OVA-specific Foxp3^+^ CD4^+^ T cells among total OVA-specific CD4^+^ T cells from the spleen and MLNs of mice treated with control antibody (RatIgG) or αIL-4, **C,** Weight change (relative to day 0 [100%]). D, H&E staining of the jejunum (n = 3 per group). These data are representative of two independent experiments.

To confirm that the suppression of excessive IL-4 production promotes the induction of aiTregs in each tissue, R23-3 mice were injected intravenously with anti-IL-4 mAb then fed the EW diet (Fig 6A). After the treatment, the percentage of aiTregs per total CD4^+^ T cells in the spleen did not differ between mice treated with anti-IL-4 mAb compared with the control antibody, whereas the percentage of aiTregs in the MLNs was incre1ased in the anti-IL-4 mAb group (Fig 6B, *P* < 0.01). However, in EW-fed R23-3 mice, anti-IL-4 treatment did not completely inhibit weight loss (Fig 6C) and the level of the inflammation was not different between the groups (Fig 6D); though the proliferation of OVA-specific CD4^+^ T cells from both the spleen and MLNs on day 7 in the anti-IL-4-treated EW-fed R23-3 mice was significantly attenuated (*P* < 0.05) compared with those of the mice that received the control-anibody (data not shown). These results confirm that excessive IL-4 production inhibited the induction of aiTregs in the MLNs, but not the spleens, of EW-fed R23-3 mice, and the decrease in the IL-4 levels increased the percentage of aiTregs in the MLNs. Therefore, we surmise that the level of IL-4 production directly regulates the induction of aiTregs in the MLNs of food allergic enteropathy.

### Recurrence of food allergic enteropathy in EW-fed OVA23-3 mice

**Fig 7:**
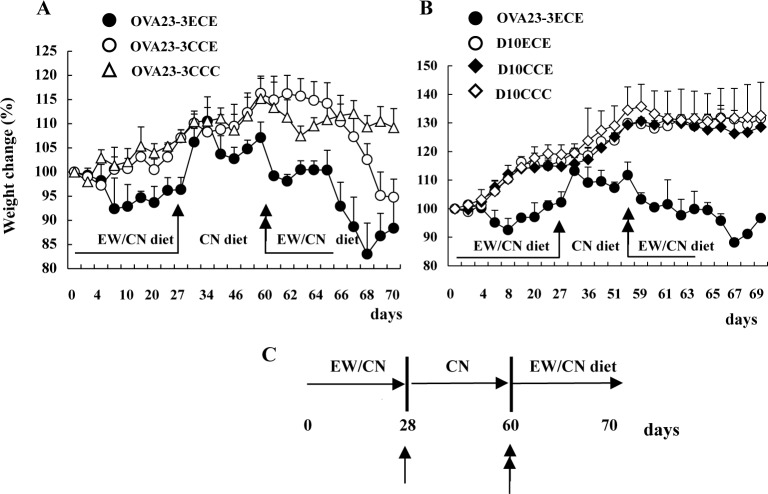
Weight changes of EW-re-fed OVA23-3 mice after given the control-diet for 1 month. **A**, Weight changes (relative to day 0 [100%]) of OVA23-3ECE mice (re-administration of EW diet after 1-month interval of CN diet; diet throughout experimental period, EW→CN→EW diet; ●, n = 3), OVA23-3CCE mice (started EW diet on day 60; diet throughout experimental period: CN→CN→EW diet; ○, n = 3), and OVA23-3CCC mice (received control [CN] diet throughout experimental period, CN→CN→CN diet; △: n = 2). **B**, Weight changes of OVA23-3ECE (●, n = 2), D10ECE (○, n = 3), D10CCE (◆, n = 3), and D10CCC (◇, n = 3) mice. **C,** Protocol for re-administrating EW diet (↑, change from EW diet to CN diet on day 28; ↑↑, re-administration of EW diet on day 60). These data are representative of two independent experiments.

To examine the effects of the regulatory activity at day 28 in EW-fed OVA23-3 and D10 mice, OVA23-3 or D10 mice were re-fed the EW diet after given the control-diet for 28 days. OVA23-3 mice that were restarted on the EW diet (that is, OVA23-3ECE mice) began to lose weight within 1 day (Fig 7A), but EW-re-fed D10 mice (D10ECE mice) demonstrated no such weight loss (Fig 7B). These results suggest that memory Th2 pathogenic T cells, which might be generated during the first round of EW feeding, responded immediately to re-administration of the EW diet in the OVA23-3 mice. During days 2 through 5 after re-starting the EW diet (days 62~66), weight was maintained without further decreases in the OVA23-3ECE group, but thereafter, continuing the EW diet led to severe weight loss. The maintenance of the weight during days 62~66 might have been due to aiTregs that were induced during the first 28-day EW-diet period. These results indicate that the hyporesponsiveness of T cells observed in the EW-fed OVA23-3 mice was due to desensitization and insufficient tolerance, whereas that of the EW-fed D10 mice was due to tolerance, even though they demonstrated immediate systemic Th2 and minor intestinal changes on day 3 of the EW diet (Fig 4C and 4D).

## Discussion

In the present study, we analyzed the immune responses to an EW diet in the two strains of mice (OVA23-3 and D10 mice), thereby established the system to explore the mechanism predominantly inducing either intestinal inflammation or tolerance to orally administered OVA. Comparing T-cell-dependent immune responses to the EW diet between these models (induction of tolerance or enteropathy) revealed differences in IL-4-associated modulation of regulatory responses between them; the IL-4 level influenced the percentage of aiTregs in the MLNs; excessive IL-4 production inhibited the generation of aiTregs, leading to the development of T cell-dependent enteropathy. Unlike the suppressive aiTregs induced in the tolerance model, our study suggested that the aiTregs induced under conditions of excessive IL-4 in the MLNs in our model of T cell-dependent food allergic enteropathy may consist of pathogenic and tolerogenic cells. In addition, independent of either tolerance or inflammation, the systemic induction of aiTregs was not markedly influenced by Th2 circumstances; IL-4 responses might contribute to establishing systemic tolerance by promoting IgG1 production.

In the MLNs of EW-fed OVA23-3 or R23-3 mice, excessive and persistent production of IL-4 attenuated the percentage of Tregs and caused enteropathy on days 7–10. Continued feeding of the EW diet until day 28 enabled the OVA23-3 and R23-3 mice to recover from the enteropathy, with a decrease in the excessive IL-4 production by OVA-specific CD4^+^ T cells and with an increase in the percentage of aiTregs.

In D10 and RD10 mice, the maintenance of lower levels of IL-4 likely effectively induced aiTregs in the MLNs [16] and prevented the mice from developing food allergic inflammation. In contrast, high levels of systemic IL-4 production earlier during the sensitization period might not influence aiTreg generation but instead promote increased serum levels of anti-OVA-specific IgG1 antibodies, which reportedly inhibit IgE-mediated reactions during oral immunotherapy [17]. Although modulating IL-4 levels might scarcely alter the systemic induction of aiTregs, IL-4 production might be diminished sufficiently to induce antibody production as well as aiTregs during systemic tolerance. Why the regulation of IL-4 production differs between the spleen and MLNs is unknown. Perhaps the IL-4 production by OVA-specific T cells and the generation of aiTregs are regulated by the original characteristics of the OVA-specific T cells [9] (Fig 2 and S2 Fig). In this regard, we found that naïve CD4^+^ T cells from RD10 mice differentiated into aiTreg cells more readily than those from R23-3 mice after OVA stimulation (S8 Fig). This result confirms that the original characteristics of the naïve CD4^+^ T cells differ between the 2 strains of mice. In addition, the conditions in each tissue as established by functions of other cells in response to the EW diet (i.e., dendritic cells or stromal cells [18–20]) may differ between the models. Furthermore, the results obtained by our anti-IL-4 mAb experiment suggest that other factors than IL-4 might contribute to the sufficient recovery from food allergic enteropathy (Fig 6). Regardless, independent of the IL-4 conditions throughout the experimental period, on day 28 of the EW diet, the hyporesponsiveness of T cells and induction of aiTregs were similar between our tolerance (D10) and food allergy (OVA23-3) models.

Therefore, to investigate whether the characteristics of the aiTregs generated under lower Th2 conditions differed from those produced during persistent Th2-mediated inflammatory conditions, we further evaluated the hyporesponsiveness of T cells acquired after 28 days of the EW diet. Re-administration of the EW diet after a 1-month interval of the control diet revealed clear differences in the hyporesponsiveness of T cells between the models (i.e., tolerance in EW-fed D10 mice but desensitization in EW-fed OVA23-3 mice) (Fig 7). These results suggest that the functions of the aiTregs acquired in the two models differ and that these different functions of (and subsequent responses by) aiTregs are influenced by the tissues in which the T cells produce excessive IL-4, the susceptibility of T cells to OVA, and the IL-4 level itself. For inducing oral tolerance, maintaining decreased levels of IL-4 in the MLNs was critical, independent of systemic IL-4 production.

Given the amplified Th2 responses and the decreased effectiveness of the aiTregs, the aiTregs likely were reprogrammed through both increased production of IL-4 and enhanced susceptibility to IL-4, such that the reprogrammed aiTregs aggravated food allergic inflammation with mast cell activation through signals from FcεRI signaling [13]. In our model, the level of serum IgE responses also seemed to correlate with aggravation of enteropathy [6]. However, the results of the present study suggests that, in EW-fed OVA23-3 and R23-3 mice, an excessive amount of IL-4 production by CD4^+^ T cells may reprogram aiTregs to become Th2-type cells; this change was mediated through OVA-specific TCR signals, not through FcεRI receptors. Without antibody production, OVA-specific CD4^+^ T cells in the MLNs of R23-3 mice receiving the EW diet for 28 days produced IL-4, with the retention of minor inflammation in their intestines. The incomplete recovery of weight loss on day 28 in the EW-fed mice strains, as well as immediate weight loss on day 1 after restarting the EW diet (on day 61 in Fig 7), suggest that the effector memory type of CD4^+^ T cells as a pathogenic memory T cell subset might abrogate recovery from and induce immediate inflammatory responses. In our present study, about 90% of induced aiTregs in the spleens and MLNs of R23-3 mice exhibited a CD62L^low^CD44^high^ phenotype (effector memory phenotype), whereas about 60% to 70% had that phenotype in the RD10 mice (S9 Fig). In EW-fed R23-3 mice, the severe inflammatory responses caused by excess IL-4 probably stimulated aiTregs to home to intestinal tissues to suppress food allergic enteropathy. Although aiTregs suppress the proliferation of T cells, they may not easily inhibit the IL-4 production of OVA-specific CD4^+^ T cells [21], and aiTregs might be reprogrammed to Th2 cells themselves in a T-cell-dependent manner and aggravate enteropathy. In addition, because a CD62L^low^CD44^high^ phenotype has been reported for colitogenic CD4^+^ memory T cells in murine colitis models [22], aiTregs that are reprogrammed as Th2-type cells might be retained as a pathogenic memory T-cell subset in the MLNs or other tissues of EW-fed OVA23-3 and R23-3 mice. In D10 and RD10 mice, we did not observe minor weight loss even after re-administration of the EW diet. Therefore, we should consider the function and phenotypes as shown in surface markers of aiTreg in R23-3 and RD10 mice in the regulation of antigen-specific Th2 cell responses and the induction of tolerance. By this analysis, we may find the way to effectively induce regulatory responses and achieve a complete tolerance during the intestinal inflammation under condition of excessive IL-4 levels.

We focused on the use of oral immunotherapy for the effective treatment of food allergy [23, 24]. In mouse models of oral immunotherapy, the transfer of Tregs effectively overcomes food allergic anaphylaxis [25]. However, the recurrent inflammatory responses in EW-fed OVA23-3 mice but not in EW-fed D10 mice suggest that the functions of aiTregs induced during inflammatory conditions must be investigated rigorously to apply Tregs in the treatment of food allergy. In addition, the site of Treg generation (i.e., the spleen or local intestinal immune tissue) might influence their function. Our present results indicate that our OVA23-3-D10 mouse model system is effective for analyzing the differences between inflammation with subsequent desensitization versus tolerance and for establishing safe and effective oral immunotherapy.

## Supporting information

S1 Text Unlike EW-fed R23-3 mice, EW-fed RD10 mice lack food allergic enteropathy. (DOCX)

S2 Text OVA-specific antibodies responses in EW-fed D10 mice and OVA23-3 mice. (DOCX)

S3 Text Percentages of Tregs (Foxp3^+^ CD4^+^ T cells) among total CD4^+^ T cells from the control-diet-fed and EW-fed OVA23-3 mice. (DOCX)

S1 File (DOCX)

S1 Fig Unlike EW-fed R23-3 mice, EW-fed RD10 mice lack food allergic enteropathy. (TIF)

S2 Fig Proliferation and cytokine production of naïve OVA-specific CD4^+^ T cells from R23-3 and RD10 mice. (TIF)

S3 Fig OVA-specific antibodies responses in EW-fed D10 and OVA23-3 mice. (TIF)

S4 Fig Proliferation of and IL-2 production by MLN and splenic CD4^+^ T cells. (TIF)

S5 Fig IL-4 and IFN-γ production by OVA-specific CD4^+^ T cells purified from R23-3 and RD10 mice. (TIF)

S6 Fig Percentage of Foxp3^+^ CD4^+^ T cells from spleen and MLNs of R23-3 and RD10 mice. (TIF)

S7 Fig Percentage of Foxp3^+^ CD4^+^ T cells among total CD4^+^ T cells from EW-fed OVA23-3 mice. (TIF)

S8 Fig Differentiation into aiTregs from naïve OVA-specific CD4^+^ T cells of R23-3 and RD10 mice against OVA stimulation. (TIF)

S9 Fig Percentage of Foxp3^+^ CD62L^low^ CD44^high^ CD4^+^ T cells among total Foxp3^+^ CD4^+^ T cells from R23-3 and RD10 mice. (TIF)
