# Supplementary material for: Critical role of intestinal interleukin-4 modulating regulatory T cells for desensitization, tolerance, and inflammation of food allergy
Source: PLoS One. 2017 Feb 24;12(2):e0172795. doi: 10.1371/journal.pone.0172795 (PMC5325285; doi:10.1371/journal.pone.0172795)
Supplement: S1 Text — (DOCX) [file pone.0172795.s001.docx]

**S1 Text.**

**Unlike EW-fed R23-3 mice, EW-fed RD10 mice lack food allergic enteropathy**

To assess intestinal enteropathy, R23-3 and RD10 mice were fed EW diet for 7 or 28 days. Histologic analysis of jejunal sections indicates that EW diet feeding for 7 days did not lead to severe morphologic changes in RD10 mice (S1A Fig, right panel of RD10 7 days EW group); by day 28 of feeding with EW diet, scattered regions of the jejunum showed minor morphologic changes, present as crypt elongation (S1A Fig, rightmost panel of RD10 28 days EW group). The ratio of villous height to crypt depth, a parameter of enteropathy (as described in Methods), did not differ significantly between EW-fed and control-diet–fed RD10 mice (S1B Fig, upper panel). In contrast to the non-inflammatory phenotype of EW-fed RD10 mice, R23-3 mice showed severe small intestinal inflammation on day 7 of EW feeding (S1A Fig, right panel of R23-3 7 days EW group), which is similar to the enteropathy found in EW-fed OVA23-3 mice (Fig 1). This response comprised a thickened muscular layer, crypt elongation, villous atrophy, and goblet cell hyperplasia. The severity of the inflammatory response in EW-fed R23-3 mice was supported by the significant decrease in the ratio of villous height to crypt depth compared with that of control-diet–fed group (S1B Fig, lower panel). These changes were largely repaired in R23-3 mice on day 28 of EW diet feeding. Although patchy regions of jejunal tissue exhibited inflammatory morphological changes comprising villous blunting and cell infiltration into the lamina propria, which are indicative of mucosal repair (S1A Fig, left panel of R23-3 28 days EW group) (6), the majority of the tissue showed normal villi (S1A Fig, right panel of R23-3 mice 28 days EW group), such that the ratio of villous height to crypt depth was similar to that of the control-diet group (S1B Fig lower panel). These changes in RD10 and R23-3 mice are consistent with the morphologic changes of EW-fed D10 and OVA23-3 mice, respectively (Fig 1B).
