# Supplementary material for: Critical role of intestinal interleukin-4 modulating regulatory T cells for desensitization, tolerance, and inflammation of food allergy
Source: PLoS One. 2017 Feb 24;12(2):e0172795. doi: 10.1371/journal.pone.0172795 (PMC5325285; doi:10.1371/journal.pone.0172795)
Supplement: S2 Text — (DOCX) [file pone.0172795.s002.docx]

**S2 Text.**

**OVA-specific antibodies responses in EW-fed D10 mice and OVA23-3 mice**

Serum OVA-specific IgG1 and IgG2a titers on day 28 of the EW diet were significantly higher in OVA23-3 than in D10 mice (*p* < 0.05). Serum OVA-specific IgE level in EW-fed D10 mice (n = 4) was considerably lower than in EW-fed OVA23-3 mice (n = 2). In another experiment, the OVA-specific IgE level in EW-fed D10 mice was not detected. The OVA-specific IgE level in the D10 mice fed the control-diet was below the limit of detection. On day 10, OVA-specific IgE was not detected in any of the OVA23-3 or D10 mice. However, the level of OVA-specific IgG1 was significantly higher in D10 than in OVA23-3 mice on day 10 (S3 Fig, *p* < 0.05), indicating greater Th2 responses were induced in EW-fed D10 mice.
