# Supplementary material for: Critical role of intestinal interleukin-4 modulating regulatory T cells for desensitization, tolerance, and inflammation of food allergy
Source: PLoS One. 2017 Feb 24;12(2):e0172795. doi: 10.1371/journal.pone.0172795 (PMC5325285; doi:10.1371/journal.pone.0172795)
Supplement: S3 Text — (DOCX) [file pone.0172795.s003.docx]

**S3 Text.**

**Percentages of Tregs (Foxp3^+^ CD4^+^ T cells) among total CD4^+^ T cells from the control-diet-fed and EW-fed OVA23-3 mice.**

In control-diet-fed and EW-fed OVA23-3 mice, the percentages of Tregs per total CD4^+^ T cells were significantly increased on day 9 during the inflammatory phase (spleen: 22.1%; MLNs: 11.2%), compared with the control-diet-fed normal phase (spleen: 7.1%; MLNs: 4.2%, *p* < 0.01). The percentages of Tregs were further significantly increased on day 28 during the recovery phase (spleen: 29.6%; MLNs: 23.5%) compared with those during the inflammatory phase (*p* < 0.01). The percentage of Tregs in the MLNs on day 28 was twice that of on day 10 (S7 Fig). The difference between the percentage of Tregs in the MLNs and those in the spleen was greater during the inflammatory phase than the recovery phase, as described in the Results section.
