# Supplementary material for: Critical role of intestinal interleukin-4 modulating regulatory T cells for desensitization, tolerance, and inflammation of food allergy
Source: PLoS One. 2017 Feb 24;12(2):e0172795. doi: 10.1371/journal.pone.0172795 (PMC5325285; doi:10.1371/journal.pone.0172795)
Supplement: S1 File — (DOCX) [file pone.0172795.s004.docx]

**S1 File.**

**Legends for supporting figures**

**S1 Fig.** **Unlike EW-fed R23-3 mice, EW-fed RD10 mice lack food allergic enteropathy.** Morphological changes in the jejunum from R23-3 and RD10 mice fed with control (CN) or EW diet (EW) were analyzed by hematoxylin and eosin staining. **A**, Representative panels from each group of mice. On day 7 of EW-diet feeding, morphological changes (crypt elongation, villous atrophy, and goblet cell hyperplasia) were present in EW-fed R23-3 mice, but not in EW-fed RD10 mice. On day 28 of EW-diet feeding, the jejunal inflammation of R23-3 mice was become patchy (left panel) with the majority of regions showing normal villi (right panel) and minor changes (right panel) were observed in scattered regions of the normal jejunum (left panel) in RD10 mice. **B**, The ratio of villous height to crypt depth (a typical parameter for monitoring morphology) in 10 randomly selected villi per mouse (*; *P* < 0.05). These data are representative of the 2 replicate experiments using 2 to 5 mice per group.

**S2 Fig. Proliferation and cytokine production of naïve OVA-specific CD4^+^ T cells from R23-3 and RD10 mice.** Naïve OVA-specific CD4^+^ T cells were purified from R23-3 (●, n = 3) and RD10 (○, n = 3) mice and stimulated with OVA (0 to 20 mg/mL). **A**, Proliferation of OVA-specific CD4^+^ T cells. **B,** IL-4 (upper panel) and IFN-γ (lower panel) levels in the culture supernatants of OVA-specific CD4^+^ T cells. These data are representative of the 2 replicate experiments.

**S3 Fig.** **OVA-specific antibodies responses in EW-fed D10 and OVA23-3 mice.** Serum OVA-specific IgG1, IgG2a and IgE from control diet (CN)-fed or EW (EW)-fed OVA23-3 or D10 mice were analyzed by ELISA. On day 10, the production of OVA-specific IgG1 and IgG2a, but not OVA-specific IgE was detected. On day 28, the all three OVA-specific antibodies were detected. Each experiment was performed three times (D10, n = 4; OVA23-3, n = 2 or 3 per experiment). These results are representative of three experiments (total numbers of mice; D10: n = 10; OVA23-3, n = 7).

**S4 Fig.** **Proliferation of and IL-2 production by MLN and splenic CD4^+^ T cells.** **A,** Proliferation of OVA-specific CD4^+^ T cells purified from EW-fed R23-3 and RD10 mice on day 7 (left panel) and 28 (right panel) of experimental period. **B,** IL-2 production of OVA-specific CD4^+^ T cells purified from EW-fed OVA23-3, D10 (left panel), R23-3, and RD10 (right panel) mice on day 28 of EW diet. Black bars and gray bars indicate the CD4^+^ T cell response from mesenteric lymph nodes (MLN) and spleen (SP) of EW-fed mice, respectively; white bars show responses of control (CN)-diet-fed mice. These results are representative of two or three experiments using EW-fed OVA23-3 (n = 4), CN-fed OVA23-3 (n = 4), EW-fed D10 (n = 4), CN-fed D10 (n = 4), CN-fed R23-3 (n = 2), EW-fed R23-3 (n = 3), CN-fed RD10 (n = 2), and EW-fed RD10 (n = 3 or 4) mice. *, *P* < 0.05; **, *P* < 0.01.

**S5 Fig. IL-4 and IFN-γ production by OVA-specific CD4^+^ T cells purified from R23-3 and RD10 mice.** CD4^+^ T cells were purified from spleens (SP) and mesenteric lymph nodes (MLN) of control-diet-fed (CN) or EW-fed (EW) R23-3 or RD10 mice. These cells were stimulated with OVA and antigen-presenting cells. IL-4 (**A**) and IFN-γ (**B**) production was detected by ELISA. Black bars (MLN) and gray bars (SP) indicate the CD4^+^ T cell responses from EW-fed mice respectively; white bars show the responses of CN-fed mice on day 7 (upper panel) or 28 (lower panel) of experimental period. These results are representative of two experiments using CN-fed R23-3 (n = 2), CN-fed RD10 (n = 2), EW-fed R23-3 (n = 3), and EW-fed RD10 (7 days, n = 3; 28 days, n = 4) mice; *, *P* < 0.05.

**S6 Fig.** **Percentage of Foxp3^+^ CD4^+^ T cells from spleen and MLNs of R23-3 and RD10 mice.** Single-cell suspensions were prepared from spleens and MLNs of R23-3 and RD10 mice fed with control (CN) or EW (EW) diet for 7 or 28 days, and Foxp3^+^ CD4^+^ T cells were detected by flow cytometry. The number shown in each plot is the ratio (given as a percentage) of Foxp3^+^ cells to CD4^+^ T cells. These data are representative of two independent experiments using CN-fed RD10 (EW0: n = 3), EW-fed RD10 (7 days: n = 3; 28 days: n = 4), CN-fed R23-3 (EW0: n = 2), and EW-fed R23-3 (7 days: n = 3; 28 days: n = 4) mice.

**S7 Fig.** **Percentages of Foxp3^+^ CD4^+^ T cells among total CD4^+^ T cells from EW-fed OVA23-3 mice.** Total CD4^+^ T cells were prepared from the spleen (SP) and mesenteric lymph nodes (MLN) of control-diet-fed (CN) or EW-fed OVA23-3 mice on day 10 (EW10) and 28 days (EW28) of experimental period. The expression of Foxp3 molecules by CD4^+^ T cells was analyzed by flow cytometry. These experiments were performed twice using OVA23-3 mice fed the CN diet (EW 0 days, n = 4) or EW diet (10 days, n = 4; 28 days: n = 4) *, *P* < 0.05; **, *P* < 0.01.

**S8 Fig.** **Differentiation into aiTreg cells from naïve CD4^+^ T cells of R23-3 and RD10 mice against OVA stimulation.** Naïve CD4^+^ T cells were purified from the spleens of R23-3 (●, n = 4) and RD10 (○, n = 6) mice. These cells were stimulated with OVA (0 to 10 mg/mL) and CD4^–^ antigen presenting cells for 72 hours, after which cells were collected and Foxp3 expression was analyzed by flow cytometry. *, *P* < 0.05

**S9 Fig. Percentage of Foxp3^+^ CD62L^low^ CD44^high^ CD4^+^ T cells among total Foxp3^+^ CD4^+^ T cells from R23-3 and RD10 mice.** Single-cell suspensions were prepared from spleens (SP) and mesenteric lymph node (MLN) of R23-3 and RD10 mice fed with EW or CN diet for 28 days. The expression of various surface molecules (CD4, Foxp3, CD62L, and CD44) was analyzed by using flow cytometry. **A,** Representative data obtained by flow cytometry of CD4^+^ Foxp3^+^ cells (upper plots) and CD62L^low^ CD44^high^ cells in CD4^+^ Foxp3^+^ cells (lower plots). The number in each lower plot indicates the percentage of CD62L^low^ CD44^high^ CD4^+^ T cells among all CD4^+^ Foxp3^+^ cells. **B,** The percentage of CD62L^low^ CD44^high^ cells among Foxp3^+^ CD4^+^ T cells (*, *P* < 0.05). These data are representative two experiments, each with 4 mice per group.
