# Supplementary figures and images for: Critical role of intestinal interleukin-4 modulating regulatory T cells for desensitization, tolerance, and inflammation of food allergy
Source: PLoS One. 2017 Feb 24;12(2):e0172795. doi: 10.1371/journal.pone.0172795 (PMC5325285; doi:10.1371/journal.pone.0172795)

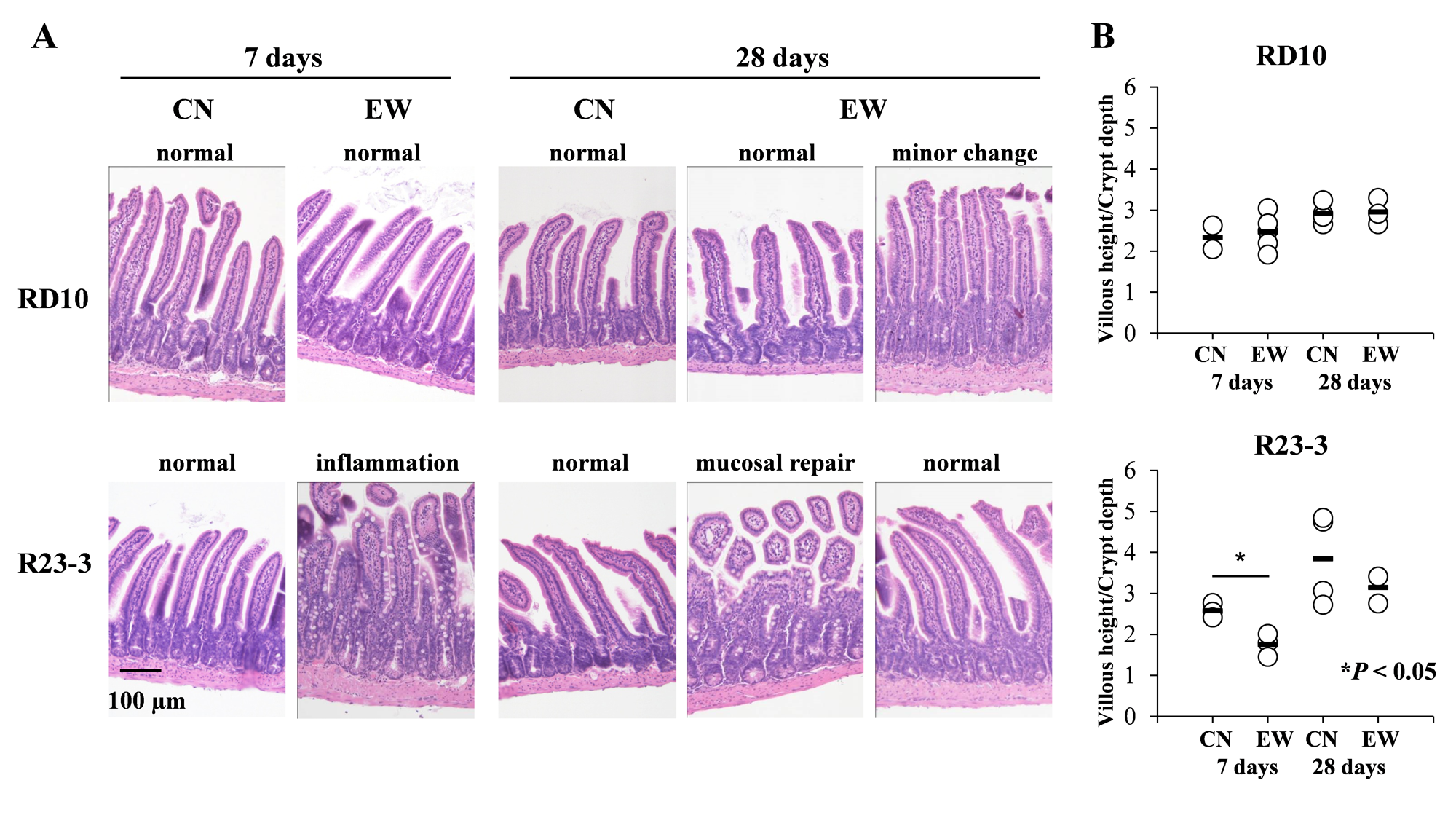

Supplement: S1 Fig — (TIF) [file pone.0172795.s005.tif]

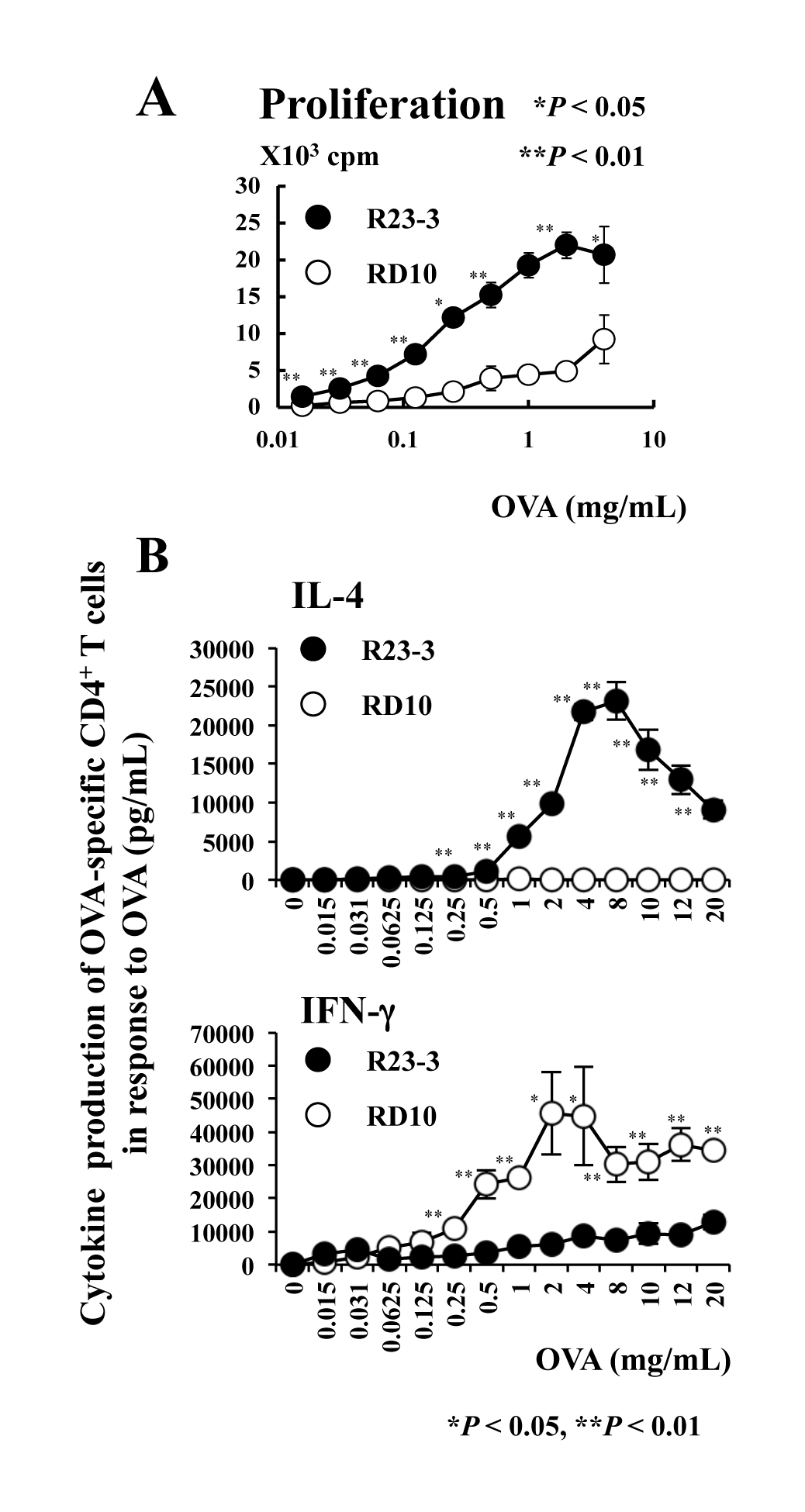

Supplement: S2 Fig — (TIF) [file pone.0172795.s006.tif]

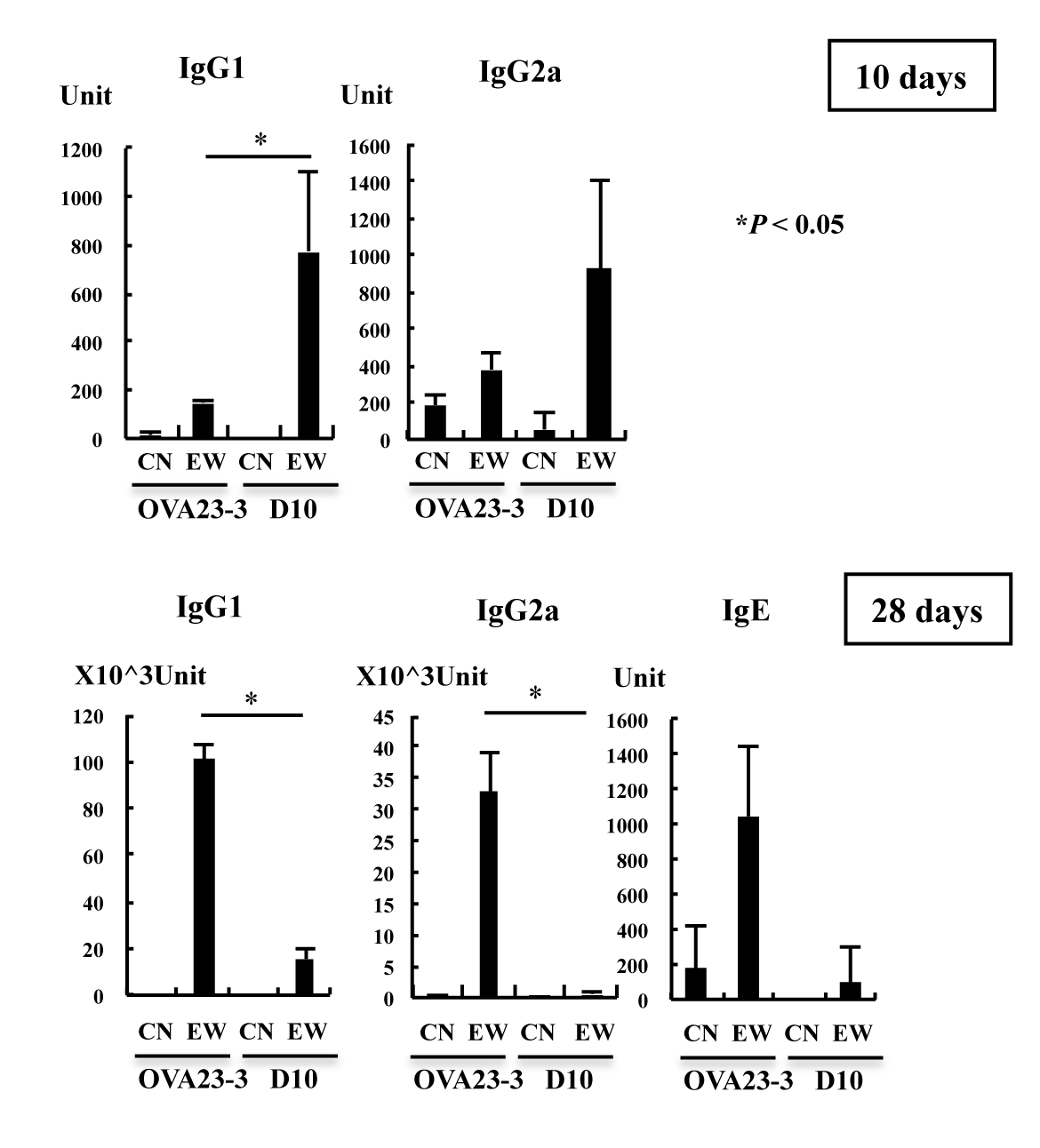

Supplement: S3 Fig — (TIF) [file pone.0172795.s007.tif]

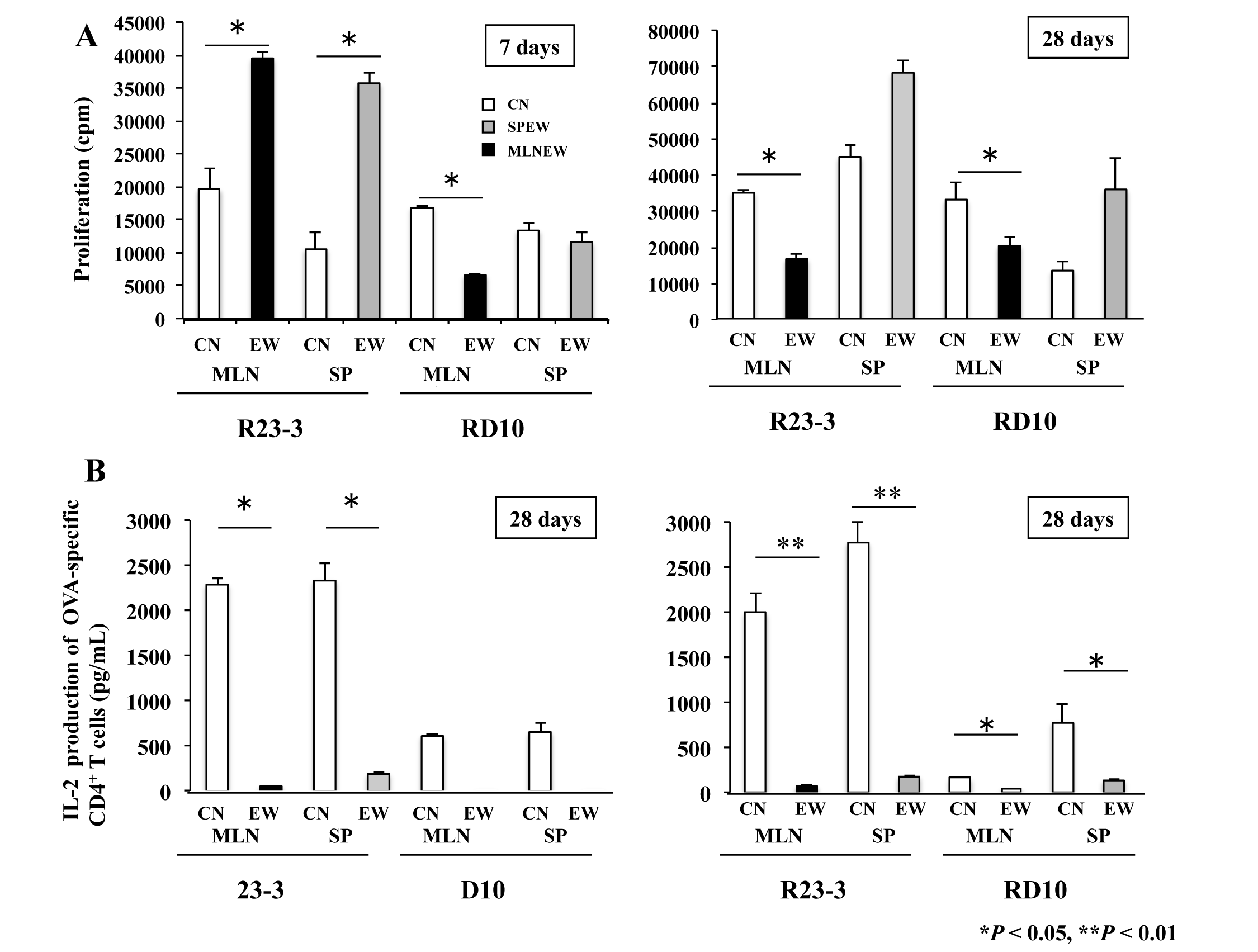

Supplement: S4 Fig — (TIF) [file pone.0172795.s008.tif]

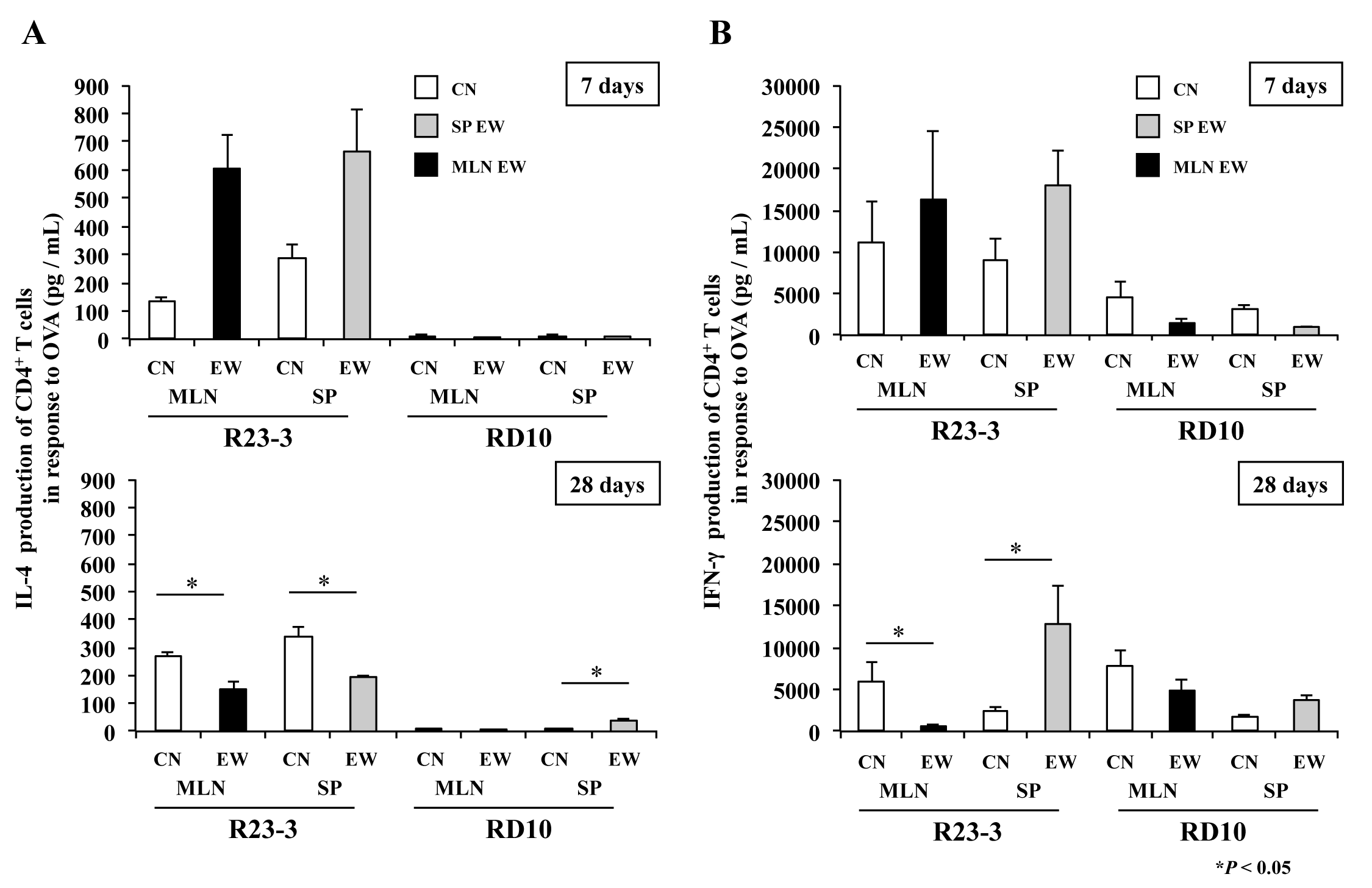

Supplement: S5 Fig — (TIF) [file pone.0172795.s009.tif]

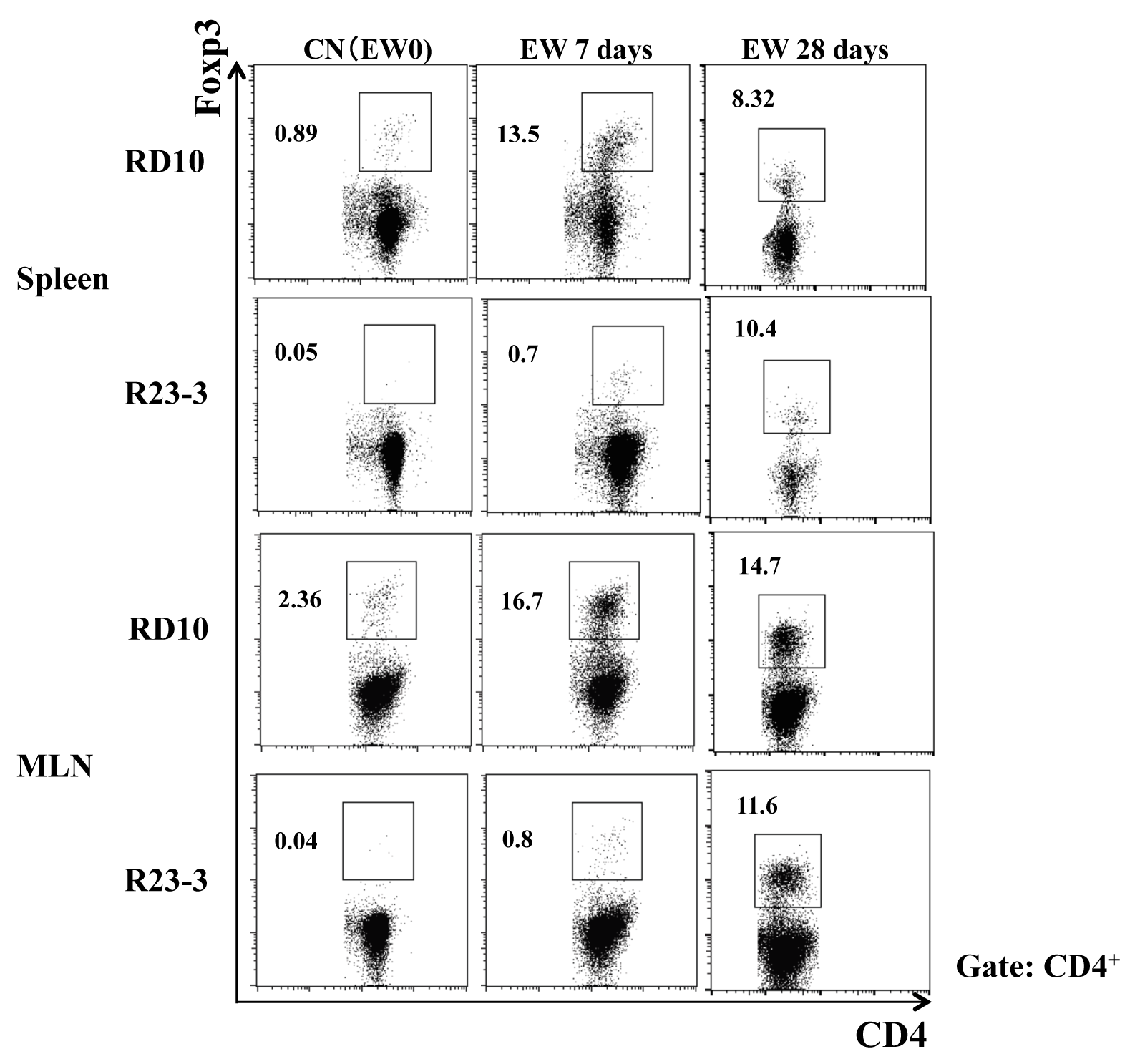

Supplement: S6 Fig — (TIF) [file pone.0172795.s010.tif]

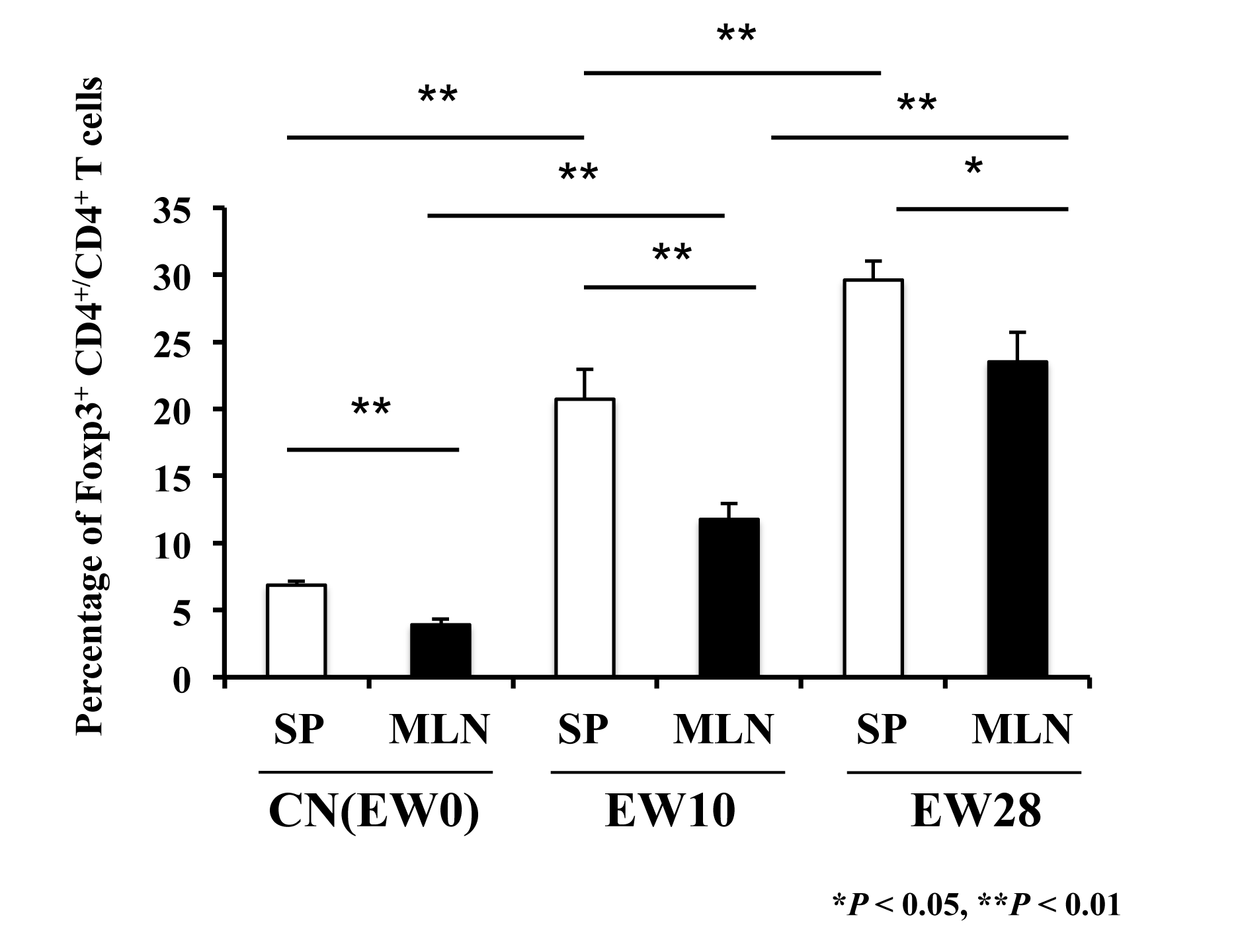

Supplement: S7 Fig — (TIF) [file pone.0172795.s011.tif]

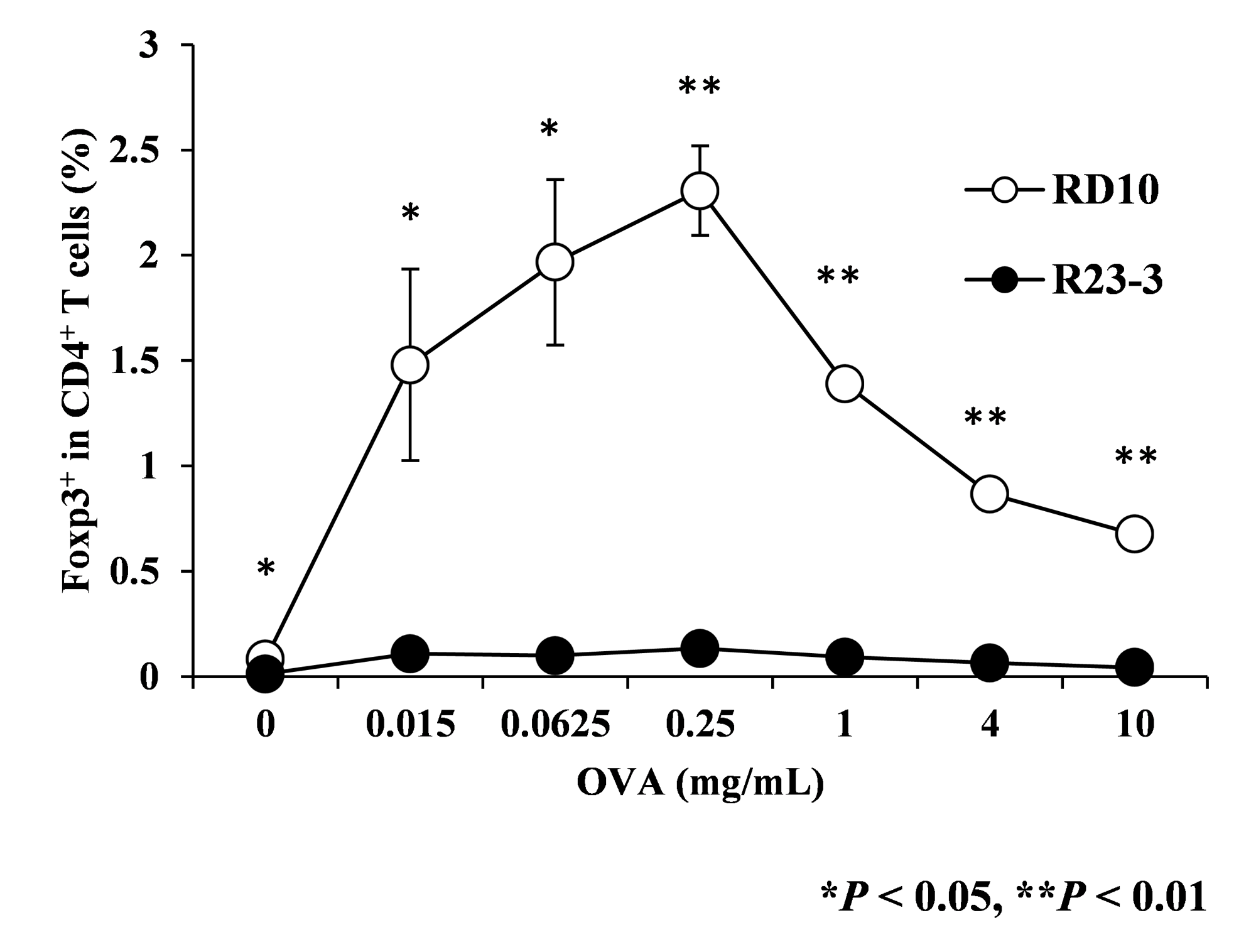

Supplement: S8 Fig — (TIF) [file pone.0172795.s012.tif]

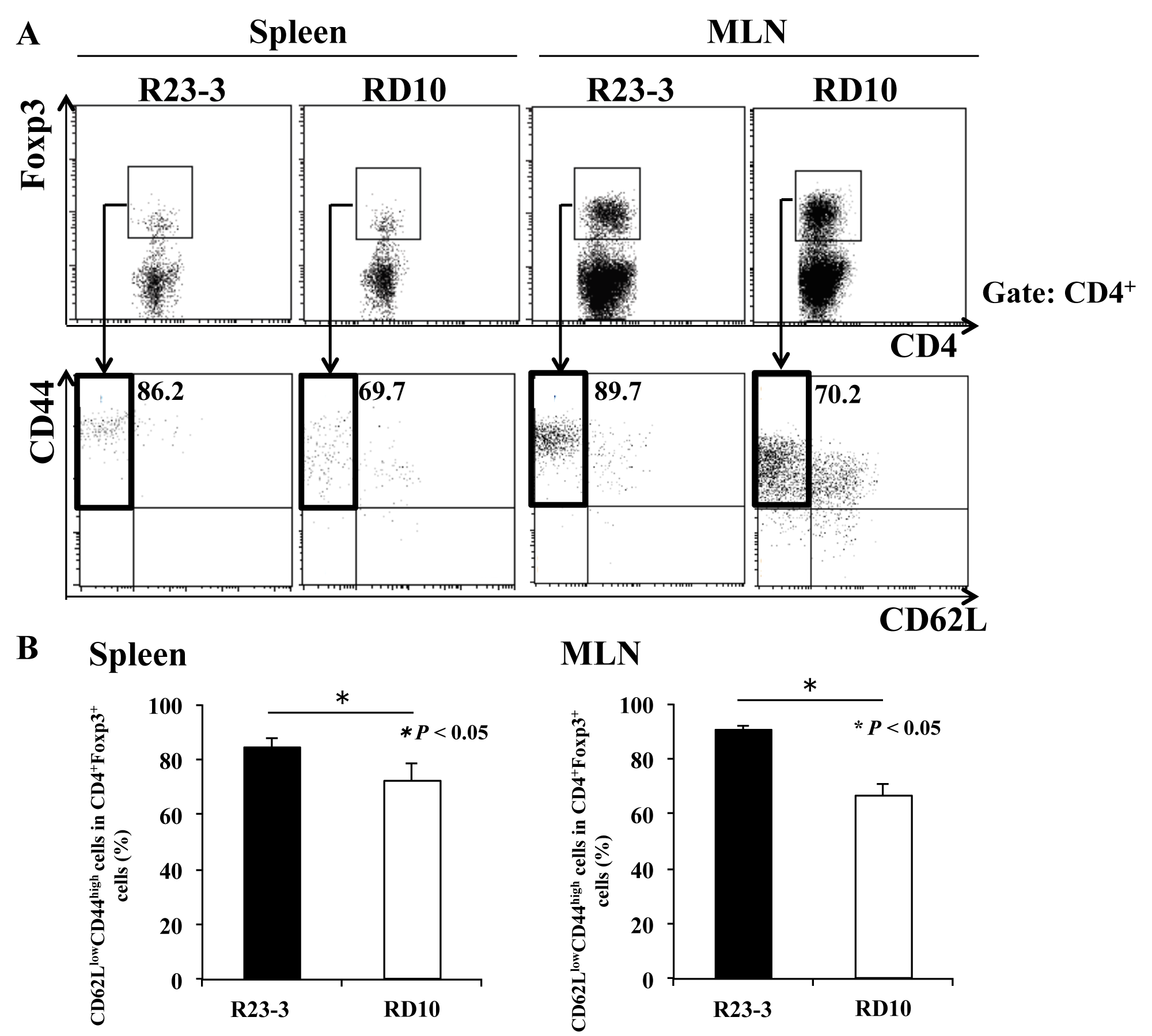

Supplement: S9 Fig — (TIF) [file pone.0172795.s013.tif]
